# COVID-19-Associated Hospitalizations Among Vaccinated and Unvaccinated Adults 18 Years or Older in 13 US States, January 2021 to April 2022

**DOI:** 10.1001/jamainternmed.2022.4299

**Published:** 2022-09-08

**Authors:** Fiona P. Havers, Huong Pham, Christopher A. Taylor, Michael Whitaker, Kadam Patel, Onika Anglin, Anita K. Kambhampati, Jennifer Milucky, Elizabeth Zell, Heidi L. Moline, Shua J. Chai, Pam Daily Kirley, Nisha B. Alden, Isaac Armistead, Kimberly Yousey-Hindes, James Meek, Kyle P. Openo, Evan J. Anderson, Libby Reeg, Alexander Kohrman, Ruth Lynfield, Kathryn Como-Sabetti, Elizabeth M. Davis, Cory Cline, Alison Muse, Grant Barney, Sophrena Bushey, Christina B. Felsen, Laurie M. Billing, Eli Shiltz, Melissa Sutton, Nasreen Abdullah, H. Keipp Talbot, William Schaffner, Mary Hill, Andrea George, Aron J. Hall, Stephanie R. Bialek, Neil C. Murthy, Bhavini Patel Murthy, Meredith McMorrow

**Affiliations:** 1 US Centers for Disease Control and Prevention COVID-19 Response, Atlanta, Georgia; 2 Public Health Service Commissioned Corps, Rockville, Maryland; 3 General Dynamics Information Technology, Atlanta, Georgia; 4 Stat-Epi Associates, Inc, Ponte Vedra Beach, Florida; 5 Field Services Branch, Division of State and Local Readiness, Center for Preparedness and Response, US Centers for Disease Control and Prevention, Atlanta, Georgia; 6 California Emerging Infections Program, Oakland; 7 Colorado Department of Public Health and Environment, Denver; 8 Connecticut Emerging Infections Program, Yale School of Public Health, New Haven; 9 Division of Infectious Diseases, School of Medicine, Emory University, Atlanta, Georgia; 10 Georgia Emerging Infections Program, Georgia Department of Public Health, Atlanta; 11 Departments of Medicine and Pediatrics, Emory School of Medicine, Atlanta, Georgia; 12 Atlanta Veterans Affairs Medical Center, Atlanta, Georgia; 13 Michigan Department of Health and Human Services, Lansing; 14 Minnesota Department of Health, St. Paul; 15 New Mexico Department of Health, Santa Fe; 16 New York State Department of Health, Albany; 17 University of Rochester School of Medicine and Dentistry, Rochester, New York; 18 Ohio Department of Health, Columbus; 19 Public Health Division, Oregon Health Authority, Portland; 20 Vanderbilt University Medical Center, Nashville, Tennessee; 21 Salt Lake County Health Department, Salt Lake City, Utah

## Abstract

**Importance:**

Understanding risk factors for hospitalization in vaccinated persons and the association of COVID-19 vaccines with hospitalization rates is critical for public health efforts to control COVID-19.

**Objective:**

To determine characteristics of COVID-19–associated hospitalizations among vaccinated persons and comparative hospitalization rates in unvaccinated and vaccinated persons.

**Design, Setting, and Participants:**

From January 1, 2021, to April 30, 2022, patients 18 years or older with laboratory-confirmed SARS-CoV-2 infection were identified from more than 250 hospitals in the population-based COVID-19–Associated Hospitalization Surveillance Network. State immunization information system data were linked to cases, and the vaccination coverage data of the defined catchment population were used to compare hospitalization rates in unvaccinated and vaccinated individuals. Vaccinated and unvaccinated patient characteristics were compared in a representative sample with detailed medical record review; unweighted case counts and weighted percentages were calculated.

**Exposures:**

Laboratory-confirmed COVID-19–associated hospitalization, defined as a positive SARS-CoV-2 test result within 14 days before or during hospitalization.

**Main Outcomes and Measures:**

COVID-19–associated hospitalization rates among vaccinated vs unvaccinated persons and factors associated with COVID-19–associated hospitalization in vaccinated persons were assessed.

**Results:**

Using representative data from 192 509 hospitalizations (see Table 1 for demographic information), monthly COVID-19–associated hospitalization rates ranged from 3.5 times to 17.7 times higher in unvaccinated persons than vaccinated persons regardless of booster dose status. From January to April 2022, when the Omicron variant was predominant, hospitalization rates were 10.5 times higher in unvaccinated persons and 2.5 times higher in vaccinated persons with no booster dose, respectively, compared with those who had received a booster dose. Among sampled cases, vaccinated hospitalized patients with COVID-19 were older than those who were unvaccinated (median [IQR] age, 70 [58-80] years vs 58 [46-70] years, respectively; *P* < .001) and more likely to have 3 or more underlying medical conditions (1926 [77.8%] vs 4124 [51.6%], respectively; *P* < .001).

**Conclusions and Relevance:**

In this cross-sectional study of US adults hospitalized with COVID-19, unvaccinated adults were more likely to be hospitalized compared with vaccinated adults; hospitalization rates were lowest in those who had received a booster dose. Hospitalized vaccinated persons were older and more likely to have 3 or more underlying medical conditions and be long-term care facility residents compared with hospitalized unvaccinated persons. The study results suggest that clinicians and public health practitioners should continue to promote vaccination with all recommended doses for eligible persons.

## Key Points

**Question:**

How do COVID-19–associated hospitalization rates compare among adults who are unvaccinated and vaccinated, and what are the risk factors for hospitalization for COVID-19 among vaccinated persons?

**Findings:**

In this cross-sectional study of US adults hospitalized with COVID-19 during January 2022 to April 2022 (during Omicron variant predominance), COVID-19-associated hospitalization rates were 10.5 times higher in unvaccinated persons and 2.5 times higher in vaccinated persons with no booster dose, respectively, compared with those who had received a booster dose. Compared with unvaccinated hospitalized persons, vaccinated hospitalized persons were more likely to be older and have more underlying medical conditions.

**Meaning:**

The study results suggest that COVID-19 vaccines are strongly associated with prevention of serious COVID-19 illness.

## Teaser

This cross-sectional study examines characteristics of COVID-19–associated hospitalizations among vaccinated persons and comparative hospitalization rates in unvaccinated and vaccinated persons.

## Introduction

As of April 30, 2022, 219.7 million people in the US had received a COVID-19 primary vaccine series, including more than 76% of the population 18 years or older. More than 100.6 million (45.8%) had also received additional or booster doses, which were recommended for people with immunosuppression in August 2021, all persons 65 years or older in September 2021, and all persons 18 years or older in November 2021.^1,2^ Data demonstrate that COVID-19 vaccines are strongly associated with prevention of COVID-19–associated hospitalization in adults, especially with the addition of a booster dose.^3,4,5^ Infections in vaccinated persons are expected,^6^ even in the setting of effective vaccines. Although most infections in vaccinated persons have been mild or asymptomatic,^6^ serious SARS-CoV-2 infections can occur in vaccinated persons.^7^ Using data from the Coronavirus Disease 2019–Associated Hospitalization Surveillance Network (COVID-NET), which represents more than 192 000 COVID-19–associated hospitalizations from January 2021 to April 2022, factors associated with hospitalizations among vaccinated persons were assessed. Population-based hospitalization rates by vaccination status were compared, including during the period when the highly transmissible B.1.1.529 (Omicron) variant of SARS-CoV-2 became the predominant circulating variant.^8^ Unlike previously published reports^9,10^ and web pages^11^ that include COVID-NET data, this study reports hospitalization rates by vaccination status and clinical and demographic characteristics of hospitalized patients, beginning with the period when vaccines first became available, and includes comparisons of unvaccinated persons, persons vaccinated with a primary series without a booster dose, and those vaccinated with a primary series and at least 1 booster dose.

## Methods

### Description and Data Collection for All COVID-NET Cases

COVID-NET is a population-based surveillance system that captures laboratory-confirmed COVID-19–associated hospitalizations in 99 counties in 14 states (California, Colorado, Connecticut, Georgia, Iowa, Maryland, Michigan, Minnesota, New Mexico, New York, Ohio, Oregon, Tennessee, and Utah); it represents approximately 10% of the US population. Hospitalized patients residing in a surveillance catchment area with a positive molecular or rapid antigen detection test result for SARS-CoV-2 during hospitalization or within 14 days before admission are included as COVID-NET cases.^12^ One site (Iowa) did not have access to reliable immunization information system (IIS) data and was excluded. Beginning in December 2021, Maryland data were also excluded from all analyses.

Demographic information, including age, race and Hispanic ethnicity, sex, hospital admission date, and evidence of a positive SARS-CoV-2 test result, are transmitted weekly on all patients, allowing calculation of population-based hospitalization rates.^12^ Race and ethnicity were categorized as Hispanic or Latino (Hispanic), non-Hispanic American Indian or Alaska Native (American Indian or Alaska Native), non-Hispanic Asian or Pacific Islander (Asian or Pacific Islander), non-Hispanic Black (Black), and non-Hispanic White (White). Race and ethnicity data were obtained from sources, including notifiable disease, laboratory, and hospital databases. In most cases, race and ethnicity were self-reported, but the source could not be confirmed in every case. This study was limited to patients 18 years or older, was reviewed and approved by the US Centers for Disease Control and Prevention (CDC) and was conducted in accordance to applicable federal law and CDC policy.^13^ This cross-sectional study is reported following the Strengthening the Reporting of Observation Studies in Epidemiology (STROBE) reporting guidelines.

### Sampling and Weighting Methods

Detailed medical record review was performed on a representative sample of patients stratified by age group and site. For sample selection, random numbers were generated and assigned to each case. Sampling weights were based on the probability of selection; sample sizes varied by surveillance month, site, and age group and were based on the total number of cases identified in each of these strata (eMethods in the Supplement).^14^

### Vaccination Definitions and Weighting of Cases With Known Vaccination Status

Being vaccinated with a primary series was defined as receiving either a second dose of a 2-dose series or 1 dose of a single-dose series 14 days or more before a positive SARS-CoV-2 test result. A patient was defined as *boosted* if they had a positive SARS-CoV-2 test result 14 days or longer after receiving an additional or booster dose of any COVID-19 vaccine on or after August 13, 2021, the date the Advisory Committee on Immunization Practices first recommended additional doses.^2^ Because the immune status of all cases is not known, an additional dose (recommended for persons with a weakened immune system) cannot be distinguished from a booster dose in this study. In this study, *vaccinated* was defined as receiving a primary series with and without a booster dose unless otherwise specified. Hospitalization rates for those vaccinated with a primary series only without a booster were compared with those vaccinated with a booster starting 14 days since at least 5% of the age-group specific population in the COVID-NET catchment area had received a booster dose. Partially vaccinated patients who had received 1 dose of a messenger RNA vaccine but had not completed a primary series were excluded.

Vaccination status for hospitalized cases and vaccine coverage for the underlying catchment area were determined by IIS data, as previously described, for all sampled COVID-NET cases.^15^ In addition to the data elements required for each case, some sites opted to collect vaccine information on all cases. With this additional information, nonsampled cases could be included in analyses regarding vaccination data. If a site did not collect vaccine information on nonsampled cases, their original sample weight was applied, and only sampled cases were included in analyses. The inclusion of sampled and nonsampled cases with known vaccination status (vaccine sample) allowed COVID-NET to retain a representative sample of all COVID-19–associated hospitalizations while allowing for more precise estimates regarding vaccine data.

### Clinical Characteristics and Outcomes Among a Weighted Sample of Vaccinated and Unvaccinated Hospitalized Patients With COVID-19

For all sampled cases, medical record abstractions were conducted using a standard case report form. Demographic information, underlying medical conditions, clinical outcomes, signs and symptoms at admission, and the likely reason for admission were compared between unvaccinated and vaccinated sampled cases (comparison sample). Underlying medical conditions were categorized into major groups (eTable 1 in the Supplement). Two physicians reviewed the reason for admission. Those patients whose reason for admission might have been incidental to COVID-19 were excluded from comparison sample analyses.^16^

Multivariable logistic regression was used to compare factors associated with hospitalizations among vaccinated and unvaccinated persons. Logistic regression was used to explore the association between vaccination status and severe COVID-19, defined as intensive care unit (ICU) admission or in-hospital death. To further account for potential confounding, a sensitivity analysis was performed using a matched propensity score analysis that matched unvaccinated and vaccinated cases in terms of demographic characteristics, underlying medical conditions, and other characteristics (eMethods in the Supplement).^17,18^

### Population-Based COVID-19–Associated Hospitalization Rates Among Unvaccinated and Vaccinated Persons

A minimum data set was collected on all cases to produce hospitalization rates (https://gis.cdc.gov/grasp/COVIDNet/COVID19_3.html). Incidence was calculated using population size from the National Center for Health Statistics’ vintage 2020 bridge-race postcensal population estimates for counties included in surveillance.^19^ To determine population-based rates of hospitalization by vaccination status per 100 000 persons 18 years or older, county-level coverage in the COVID-NET catchment area was estimated using population denominators. Vaccination status was classified as described previously using the vaccine sample. Given that the number of unvaccinated and vaccinated persons in the underlying population changed weekly, incidence (cases per 100 000 person-weeks) was calculated by dividing the total number of unvaccinated hospitalized persons by the sum of unvaccinated persons in the underlying population each week; the same method was used for incidence calculations in vaccinated persons with and without a booster dose. Incidence rate ratios and 95%CIs were calculated.

The Delta and Omicron variants became the predominant circulating variants during July 2021 and late December 2021, respectively.^8^ Because vaccination coverage and circulating variants potentially are associated with vaccine effectiveness, cumulative rate ratios are presented monthly and also in intervals (January-June [pre-Delta] and July-December 2021 [Delta] and January-April 2022 [Omicron]). A continuity correction has been applied to the denominators by capping the percentage of population vaccination coverage at 95%, which assumes that at least 5% of each age group would always be unvaccinated in each jurisdiction.^20^ This correction ensures a reasonable denominator for the unvaccinated population that would prevent hospitalization rates from growing unrealistically large because of potential overestimates of vaccination coverage. Rates were calculated for all cases that met the case definition regardless of reason for admission; overall rates for those 18 years or older were standardized to the underlying population. Rates by booster dose status were presented beginning with the date starting 14 days after at least 5% of the age group–specific population of the catchment area had received a booster dose; these were the weeks ending November 27, November 16, and October 16, 2021, for those aged 18 to 49 years, 50 to 64 years, and 65 years or older, respectively.

Limited COVID-NET clinical and hospitalization rate data by vaccination status are publicly available,^10,11^ and a recent report compared recent hospitalization rates in unvaccinated adults with those who had received a primary series plus a booster dose for a single time point.^9^ This article examined hospitalization rates and characteristics of hospitalized patients by vaccination status from the period when vaccines first became available and also compared rates among those who received a primary series with and without a booster dose using data through April 2022; similar COVID-NET analyses covering this extended period were not included in previously published data sources. An early version of the non–peer-reviewed manuscript with data through July 2021 was posted on a preprint server on August 29, 2021,^21^ before the widespread availability of booster doses.

### Statistical Analysis

Data from all cases hospitalized with laboratory-confirmed COVID-19 with linked IIS data were used to describe the vaccination status of hospitalized cases by age, sex, race and Hispanic ethnicity, and admission month. Multivariable models included a priori age groups, sex, race and Hispanic ethnicity, and long-term care facility (LTCF) residency; models incorporated clustering by site to account for geographic differences. Other variables with *P* values less than .10 in bivariate analyses were included in the multivariable analyses. Model fit was assessed with quasilikelihood within independence model criterion. A log-linked Poisson generalized estimating equations regression was used to generate adjusted risk ratios (aRRs) and 95% CIs. Data were analyzed using SAS survey procedures to account for sampling weights. Unweighted case counts and weighted percentages are presented unless otherwise noted. Proportions with 95% CIs are presented for binary measures and medians with interquartile ranges for continuous measures. Taylor series linearization methods were used for variance estimation.^22^ All analyses were conducted using SAS (version 9.4; SAS Institute).

## Results

**Figure 1.**
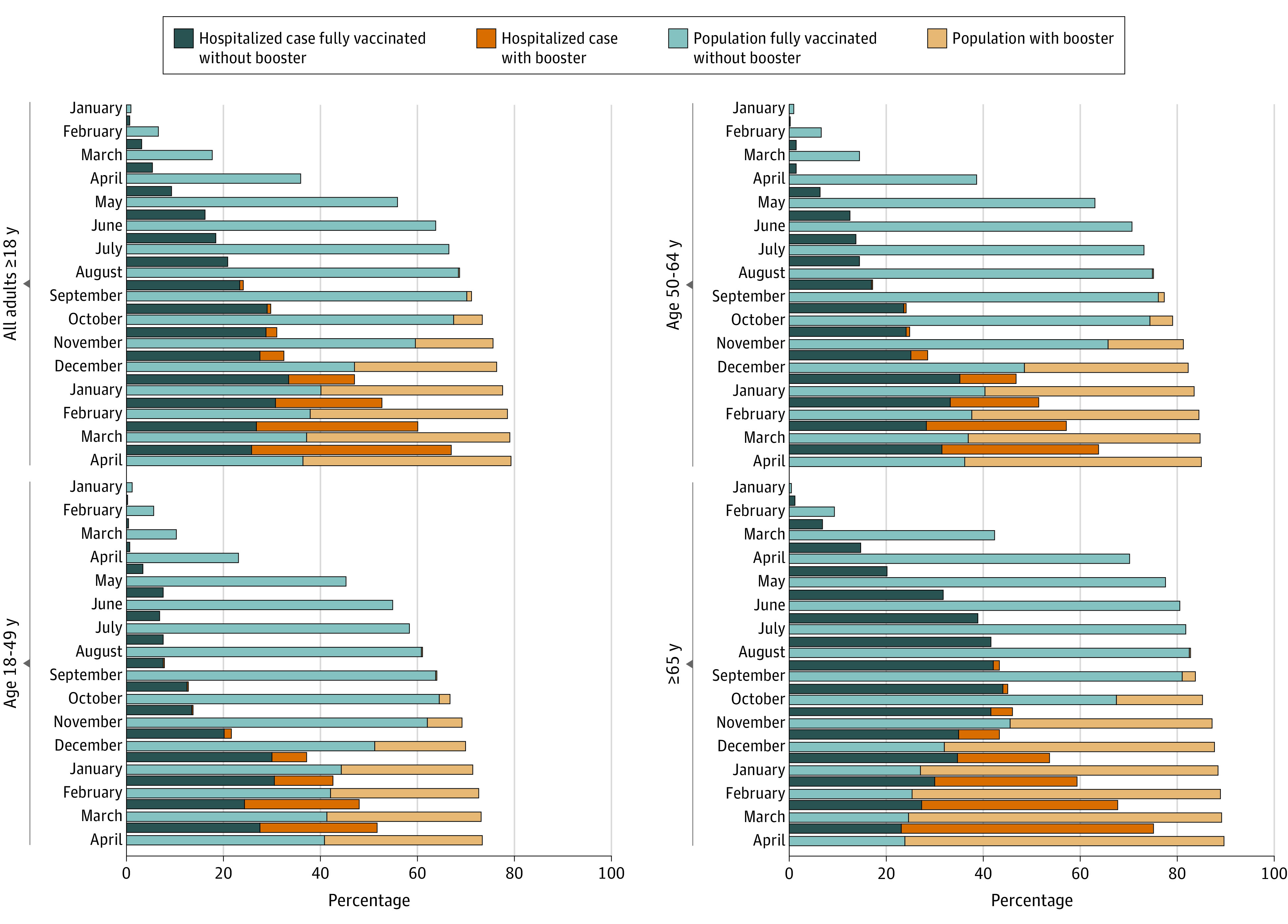
Proportion of Adults in the COVID-19–Associated Hospitalization Surveillance Network (COVID-NET) Catchment Area and Adults 18 Years or Older With COVID-19–Associated Hospitalizations Admitted January 1, 2021, to April 30, 2022, Vaccinated With a Primary Series, With and Without a Booster Dose,^a^ by Age Group and Month of Admission, COVID-NET, 13 States^b^ ^a^Because the immune status of all cases was not known, an additional dose (recommended for persons with a weakened immune system) cannot be distinguished from a booster dose. This is a relevant consideration because vaccines can be less effective in persons with a weakened immune system. Additional doses described as booster doses in the figure became available to the general public on August 13th. ^b^California, Colorado, Connecticut, Georgia, Maryland (data excluded beginning December 4, 2021), Michigan, Minnesota, New Mexico, New York, Ohio, Oregon, Tennessee, and Utah.

During January 1, 2021, to April 30, 2022, 192 509 laboratory-confirmed COVID-19–associated hospitalized cases in those 18 years or older were identified in COVID-NET, among whom a representative sample of 146 937 (76%) had vaccination data linked to state IIS (vaccine sample). Among those with known vaccination status, 98 243 (69.2%) were unvaccinated; 39 353 (24.5%) were vaccinated with a primary series, among whom 8796 (22%) were boosted (eFigure and eTable 2 in the Supplement). The monthly number and proportion of hospitalized cases that were vaccinated increased from 2 (<0.1%) in January 2021 to 2239 (67.0%) in April 2022, including 75.0% of patients 65 years or older in that month. The proportion of the vaccinated population in the underlying COVID-NET catchment area increased from 0.9% to 79.3% during the same period, including 89.7% in those 65 years or older (Figure 1).

Among a representative sample of 14 164 hospitalized patients 18 years or older with medical record review, the following were excluded: 921 (5.8%) were partially vaccinated, 16 (0.1%) had unknown vaccination status, 184 (1.2%) had incomplete data, and 1916 (13%) were likely admitted because of non–COVID-19–related reasons (eFigure in the Supplement; see eTable 3 in the Supplement for the likely reason for admission by vaccination status).

The comparison sample was restricted to the remaining 11 127 patients whose reason for admission was likely associated with COVID-19. Among these patients, the median (IQR) age was 61 (49-74) years (5368 [48.3%] women; 160 [1.3%] American Indian or Alaska Native, 429 [4.7%] Asian or Pacific Islander, 2230 [24.9%] Black, 1531 [12.6%] Hispanic or Latino, and 6342 [51.4%] White individuals). This comparison sample included 8575 unvaccinated and 2552 vaccinated patients, among whom 491 (21%) were boosted (Table 1). Among vaccinated cases, the median time from most recent vaccine dose until hospital admission was 180 days (IQR, 103-246) (eTable 4 in the Supplement). Vaccinated cases were older and more likely to be White and LTCF residents compared with unvaccinated cases (Table 1). In addition, vaccinated cases were more likely to have immunosuppression compared with unvaccinated cases (560 [23.3%] vs 877 [10.8%], respectively; *P* < .001), as well as more likely to have 3 or more underlying medical conditions (1926 [77.8%] vs 4124 [51.6%], respectively; *P* < .001). Compared with vaccinated cases without a booster dose, boosted cases were more likely to have an immunosuppressive condition (144 [32.5%] vs 198 [19.9%]; *P* = .001) and rheumatologic or autoimmune disease (77 [19.1%] vs 107 [12.3%]; *P* = .03) (eTable 4 in the Supplement). Among the 1550 vaccinated patients hospitalized from October 2021 to April 2022, 487 (27.5%) were boosted, including 159 of 258 (70.4%) in April 2022. When the 342 vaccinated patients (23.4%) with immunocompromising conditions were excluded, 343 patients (28.4%) had received a booster dose, including 120 of 197 (61%) of those in April 2022 (data not shown).

**Table 1.** Characteristics by Vaccination Status for Adults Hospitalized With Laboratory-Confirmed SARS-CoV-2, January 2021 to April 2022^a^

Category	Adults ≥18 y (January 2021-April 2022)				Vaccinated adults ≥18 y (October 2021-April 2022)^b^			
	Total, No. (%)	No. (weighted %)^b^		*P* value^c^	Total	No. (weighted %)^b^		*P* value^c^
		Unvaccinated	Vaccinated with a primary series with and without booster			Vaccinated without booster	Vaccinated with booster	
Total	11 127	8575 (74.6)	2552 (25.4)		1550	1063 (72.5)	487 (27.5)	
Vaccinated without booster^b^	NA	NA	2061 (78.6)	NA	1063	1063 (100)	0	NA
Vaccinated with booster^b^	NA	NA	491 (21.4)		487	0	487 (100)	
Age group, median (IQR), y	61 (49-74)	58 (46-70)	70 (58-80)		70 (57-89)	69 (57-79)	73 (62-80)	
18-49	3220 (25.2)	2850 (29.9)	370 (12.4)	<.001	273	214 (15.0)	59 (10.2)	.01
50-64	3988 (29.8)	3246 (32.5)	742 (21.9)		509	358 (24.8)	151 (17.2)	
≥65	3919 (44.7)	2479 (37.6)	1440 (65.7)		768	491 (60.2)	277 (72.6)	
Sex								
Female	5368 (48.3)	4126 (48.5)	1242 (47.7)	NA	746	523 (50.5)	223 (42.0)	NA
Male	5759 (51.7)	4449 (51.5)	1310 (52.3)	.64	804	540 (49.5)	264 (58.0)	.04
Race and ethnicity^d^								
American Indian or Alaska Native	160 (1.3)	127 (1.3)	33 (1.4)	<.001	20	14 (1.9)	6 (0.5)	<.001
Asian or Pacific Islander	429 (4.7)	343 (4.8)	86 (4.5)		54	30 (3.7)	24 (5.4)	
Black	2230 (24.9)	1863 (27.3)	367 (17.9)		227	182 (20.4)	45 (11.8)	
Hispanic or Latino	1531 (12.6)	1297 (13.8)	234 (9.3)		140	105 (10.2)	35 (7.5)	
White	6342 (51.4)	4595 (48.1)	1747 (61.2)		1061	695 (55.7)	366 (72.9)	
Other/unknown^e^	435 (5.0)	350 (4.8)	85 (5.7)		48	37 (8.1)	11 (1.9)	
Period (row %)								
Pre-Delta (January 2021-June 2021)	4819 (34.7)	4486 (96.6)	333 (3.4)	<.001				<.001
Delta (July 2021-December 2021)	4369 (37.5)	3216 (73.3)	1153 (26.7)		484	453 (91.7)	31 (8.3)	
Omicron (January 2022-April 2022)	1939 (27.8)	873 (48.8)	1066 (51.2)		1066	610 (65.2)	456 (34.8)	
LTCF residence^f^	604 (6.8)	253 (4.5)	351 (13.8)	<.001	206	122 (12.8)	84 (17.3)	.120
No. of underlying conditions^g^								
0	1143 (9.3)	1043 (11.4)	100 (3.2)	<.001	68	51 (2.8)	17 (4.2)	.63
1	2003 (15.9)	1779 (18.8)	224 (7.5)		146	114 (7.8)	32 (6.3)	
2	1931 (16.5)	1629 (18.2)	302 (11.5)		182	123 (11.8)	59 (10.5)	
≥3	6050 (58.3)	4124 (51.6)	1926 (77.8)		1154	775 (77.7)	379 (79.0)	
Outcomes								
ICU admission	2466 (21.1)	1961 (21.7)	505 (19.5)	.13	286	206 (17.7)	80 (19.0)	.70
In-hospital death	1018 (9.9)	802 (9.9)	216 (10.1)	.89	121	91 (9.7)	30 (10.1)	.88
Had any COVID-19–related symptom	10 524 (94.5)	8227 (95.8)	2297 (90.7)	<.001	1391	980 (92.1)	411 (85.2)	.01
Median length of stay (IQR), d	4.5 (2.2-9.2)	4.6 (2.3-9.3)	4.3 (1.9-8.9)	<.001	4 (1.8-8.9)	4 (1.8-8.8)	3.9 (1.8-9.1)	<.001

Abbreviations: COVID-NET, COVID-19–Associated Hospitalization Surveillance Network; ICU, intensive care unit; LTCF, long-term care facility; NA, not applicable.

^a^ California, Connecticut, Colorado, Georgia, Maryland (data excluded beginning December 4, 2021), Michigan, Minnesota, New Mexico, New York, Ohio, Oregon, Tennessee, and Utah. The analysis was restricted to those with COVID-19 as a likely reason for admission. Note that column percentages are shown except where row percentages are indicated.

^b^ Unvaccinated: population-based rates of COVID-19–associated hospitalizations among persons with a positive SARS-CoV-2 test result who had no record of receiving any COVID-19 vaccine. Vaccinated: population-based rates of COVID-19–associated hospitalizations among persons with a positive SARS-CoV-2 test result collected 14 days or longer after vaccination with a primary series, defined as either the second dose of a 2-dose vaccine series or after 1 dose of a single-dose vaccine. When not otherwise specified, vaccinated persons include those who may have received additional or booster doses. Vaccinated without booster dose: population-based rates of COVID-19–associated hospitalizations among vaccinated persons who have received a primary series and who have not received an additional or booster dose. This includes those eligible and not yet eligible for an additional or booster dose. Vaccinated with booster dose: population-based rates of COVID-19-associated hospitalizations among persons vaccinated with a primary series who have received an additional or booster dose on or after August 13, 2021, with a positive SARS-CoV-2 test result collected 14 days or longer after receipt of an additional or booster dose. Because the immune status of all cases is not known, an additional dose (recommended for persons with a weakened immune system) cannot be distinguished from a booster dose. This is a relevant consideration because vaccines can be less effective in persons with a weakened immune system.

^c^ Statistical significance for univariate analyses was determined as *P* < .10.

^d^ Data on race and ethnicity were categorized as follows: Hispanic or Latino, Non-Hispanic American Indian or Alaska Native, Non-Hispanic Asian or Pacific Islander, non-Hispanic Black, non-Hispanic White, and Other/Unknown. If ethnicity was unknown (8% of cases), non-Hispanic ethnicity was assumed.

^e^ Includes multiracial (53 [0.5%]) and unknown race (382 [4.5%]).

^f^ Long-term care facility residence was defined as residence in rehabilitation facilities, assisted living/residential care, group homes, nursing homes, skilled nursing facilities, LTCFs, long-term acute care hospitals, residential care facilities, or other long-term care facilities.

^g^ Overall condition categories as defined in eTable 1 in the Supplement.

On multivariable analysis, older patients, LTCF residents, and those who had immunosuppression or with underlying obesity, chronic lung disease, kidney disease, neurologic disease, or rheumatologic or autoimmune disease were more likely to be vaccinated compared with younger patients or those without those specific conditions. Black and Hispanic patients were less likely to be vaccinated compared with White patients (Table 2).

**Table 2.** Multivariable Model^a^ Assessing Factors Associated With Vaccination Status in Hospitalized Adults With Laboratory-Confirmed SARS-CoV-2, January 2021 to April 2022^b^

Characteristic	No. (weighted %)		Unadjusted RR (95% CI)	aRR (95% CI)	*P* value
	Unvaccinated	Vaccinated with a primary series, with and without a booster			
Age, y					
18-49	2850 (30)	370 (12)	1 [Reference]	1 [Reference]	NA
50-64	3246 (33)	742 (22)	1.50 (1.35-1.67)	1.25 (1.12-1.39)	<.001
≥65	2479 (38)	1440 (66)	3.01 (2.5-3.63)	1.73 (1.42-2.1)	<.001
Sex					
Female	4126 (48.5)	1242 (47.7)	1 [Reference]	1 [Reference]	NA
Male	4449 (51.5)	1310 (52.3)	1.03 (0.95-1.11)	1.01 (0.94-1.08)	.83
Race and ethnicity^c^					
American Indian or Alaska Native	127 (1.3)	33 (1.4)	0.89 (0.64-1.23)	1.21 (0.81-1.79)	.36
Asian or Pacific Islander	343 (4.8)	86 (4.5)	0.81 (0.62-1.05)	0.81 (0.66-1)	.05
Black	1863 (27.3)	367 (17.9)	0.6 (0.46-0.8)	0.8 (0.68-0.93)	.004
Hispanic or Latino	1297 (13.8)	234 (9.3)	0.62 (0.49-0.77)	0.84 (0.74-0.96)	.01
White	4595 (48.1)	1747 (61.2)	1 [Reference]	1 [Reference]	NA
Other/unknown^d^	350 (4.8)	85 (5.7)	0.97 (0.76-1.23)	1.03 (0.84-1.26)	.76
In long-term care facility^e^	253 (4.5)	351 (13.8)	2.14 (1.62-2.84)	1.28 (1.05-1.56)	<.001
Underlying medical condition					
Obesity^f^	4330 (47.9)	1038 (38)	0.74 (0.66-0.82)	0.91 (0.84-0.99)	.03
Diabetes^g^	2455 (31.4)	993 (40.2)	1.33 (1.2-1.47)	1.06 (0.97-1.16)	.23
Chronic lung disease	3953 (48.2)	1781 (70)	2.01 (1.74-2.32)	1.29 (1.18-1.41)	<.001
Cardiovascular disease^h^	2379 (31.6)	1365 (53.9)	1.97 (1.7-2.28)	1.05 (0.95-1.17)	.34
Neurologic disease	1157 (14.8)	726 (30.2)	1.87 (1.62-2.17)	1.27 (1.17-1.37)	<.001
Kidney disease	992 (14.4)	687 (30.1)	1.91 (1.75-2.09)	1.27 (1.15-1.42)	<.001
Immunosuppressive condition	877 (10.8)	560 (23.3)	1.86 (1.66-2.09)	1.46 (1.33-1.6)	<.001
Gastrointestinal or liver disease	795 (9.3)	355 (12.9)	1.3 (1.09-1.55)	0.95 (0.78-1.15)	.58
Blood disorder	269 (3)	170 (7)	1.79 (1.43-2.25)	1.18 (0.97-1.43)	.09
Rheumatologic or autoimmune disease	465 (5.9)	309 (13.5)	1.85 (1.62-2.1)	1.13 (1.01-1.27)	.04

Abbreviations: aRR, adjusted risk ratio; COVID-NET, COVID-19–Associated Hospitalization Surveillance Network; NA, not applicable; RR, risk ratio.

^a^ Log-linked Poisson regression using generalized estimating equations clustered on site with exchangeable covariance structure.

^b^ California, Connecticut, Colorado, Georgia, Maryland (data excluded beginning December 4, 2021), Michigan, Minnesota, New Mexico, New York, Ohio, Oregon, Tennessee, and Utah. The analysis was restricted to those with COVID-19 as a likely reason for admission.

^c^ Data on race and ethnicity were categorized as follows: Hispanic ethnicity, Non-Hispanic American Indian or Alaska Native, Non-Hispanic Asian or Pacific Islander, non-Hispanic Black, non-Hispanic White, and Other/Unknown. If ethnicity was unknown (8% of cases), non-Hispanic ethnicity was assumed.

^d^ Includes multiracial (53 [0.5%]) and unknown race (382 [4.5%]).

^e^ Long-term care facility residence was defined as residence in rehabilitation facilities, assisted living/residential care, group homes, nursing homes, skilled nursing facilities, long-term care facilities, long-term acute care hospitals, residential care facilities, or other long-term care facilities.

^f^ Obesity is defined as calculated body mass index (calculated as weight in kilograms divided by height in meters squared) of 30 or greater, and if body mass index is missing, by *International Classification of Diseases* discharge diagnosis codes.

^g^ Diabetes includes type I and type 2 diabetes.

^h^ Cardiovascular disease excludes hypertension.

The proportion of vaccinated persons admitted to the ICU was similar to that among unvaccinated persons (505 [19.5%] vs 1961 [21.7%], respectively; *P* = .13), as were proportions for in-hospital death (216 [10.1%] vs 802 [9.9%], respectively; *P* = .89). Median length of stay in vaccinated persons was shorter (median, 4.3 days [IQR, 1.9-8.9] vs 4.6 days [IQR 2.3-9.3], respectively) (Table 1). On multivariable analysis, vaccination was not significantly associated with a reduced risk of severe disease (ie, ICU admission or death) (aRR, 0.83; 95% CI, 0.65-1.07; *P* = .16) (eTable 5 in the Supplement). The sensitivity analysis using the propensity score–matched cohort included 2000 vaccinated and 2000 unvaccinated patients (eTable 6 in the Supplement). Results from the analysis of this cohort were similar to the primary model; vaccination was not significantly associated with reduced risk of severe disease (aRR, 0.80; 95% CI, 0.59-1.10; *P* = .16; full model not shown).

### Population-Based Rates of COVID-19–Associated Hospitalization by Vaccination Status

**Figure 2.**
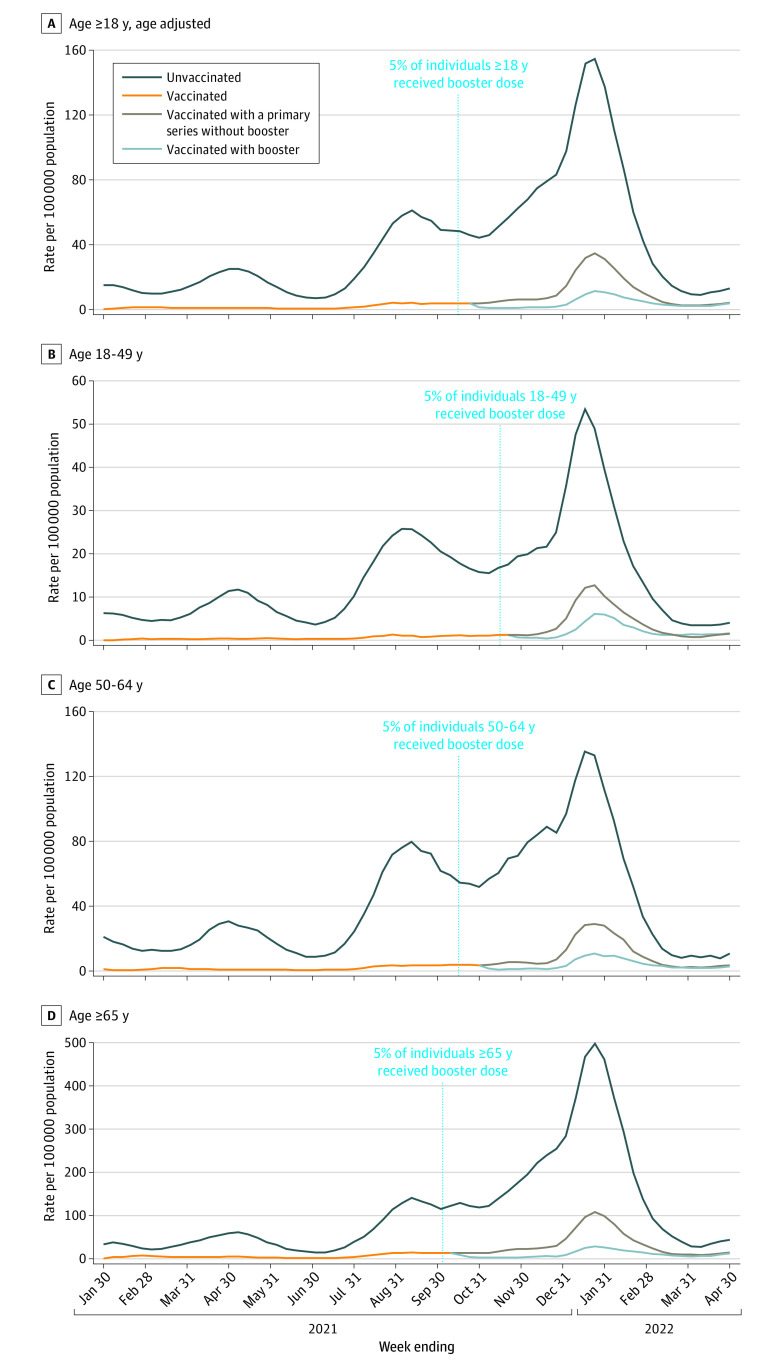
Three-Week Moving Average Population-Based Rates^a^ of COVID-19–Associated Hospitalizations Among Unvaccinated and Vaccinated (With and Without a Booster Dose)^b^ Adults 18 Years or Older Admitted January 30, 2021,^c^ to April 30, 2022, by Week of Admission, COVID-19–Associated Hospitalization Surveillance Network (COVID-NET), 13 States^d^ Data shown for individuals vaccinated with a booster for the following dates: adults 18 years and older (age adjusted), October 30, 2021, to April 30, 2022 (A), age 18 to 49 years, November 27, 2021, to April 30, 2022 (B), age 50 to 64 years, November 6, 2021, to April 30, 2022 (C), and 65 years or older, October 16 to April 30, 2022 (D).^e^ ^a^Patients with laboratory-confirmed COVID-19–associated hospitalizations per 100 000 population. ^b^Unvaccinated: persons with a positive SARS-CoV-2 test who had no record of receiving any COVID-19 vaccine. Vaccinated: persons with a positive SARS-CoV-2 test collected 14 days or more after vaccination with a primary series, defined as either the second dose of a 2-dose vaccine series or after 1 dose of a single-dose vaccine. When not otherwise specified, vaccinated persons include those who may have received additional or booster doses. Vaccinated without a booster dose: persons who have received a primary series and who have not received an additional or booster dose. This includes those eligible and not yet eligible for an additional or booster dose. Vaccinated with a booster dose: persons vaccinated with a primary series and an additional or booster dose on or after August 13, 2021, with a positive SARS-CoV-2 test collected 14 days or more after receipt of an additional or booster dose. Because the immune status of all cases is not known, an additional dose (recommended for persons with a weakened immune system) cannot be distinguished from a booster dose. ^c^The week ending January 30, 2021, is the earliest an individual could be considered to have completed a primary series based on the approval of the first COVID-19 vaccines in December 2020. ^d^California, Colorado, Connecticut, Georgia, Maryland (data excluded beginning December 4, 2021), Michigan, Minnesota, New Mexico, New York, Ohio, Oregon, Tennessee, and Utah. ^e^Period of data based on when 14 days have passed because at least 5% of the age group-specific population of the COVID-NET surveillance catchment area had received an additional or booster dose.

Monthly hospitalization rates ranged from 3.5 (95% CI, 3.3-3.8) times higher (April 2022) to 17.7 (95% CI, 16.3-19.2) times higher (May 2021) in unvaccinated persons compared with vaccinated persons regardless of booster dose status (Figure 2, A-D; eTable 7 in the Supplement). For July 2021 to December 2021 (Delta period) and January to April 2022 (Omicron period), cumulative hospitalization rate ratios in unvaccinated persons compared with vaccinated persons, regardless of booster dose status, were 12.2 (95% CI, 12.0-12.4) and 6.8 (95% CI, 6.6-6.9) for all adults 18 years or older, respectively (eTable 7 in the Supplement). From January to April 2022, rates were 10.5 (95% CI, 10.2-10.8) and 2.5 (95% CI, 2.2-2.8) times higher in unvaccinated persons and vaccinated persons with no booster dose, respectively, compared with those who had received a booster dose (data not shown).

## Discussion

Using data from a representative sample of more than 192 000 COVID-19–associated hospitalizations, population-based rates of COVID-19-associated hospitalization were approximately 10.5 times higher in unvaccinated adults compared with adults vaccinated with a primary series and a booster dose during January to April 2022, when the Omicron variant was predominant. This suggests that COVID-19 vaccines continue to effectively prevent hospitalizations in all adults. COVID-19 vaccination is an essential tool for preventing morbidity and mortality from COVID-19. A greater proportion of hospitalized cases among vaccinated persons occurred in individuals with medical fragility who were older, more likely to reside in LTCFs, and have 3 or more underlying medical conditions, including immunosuppressive conditions.

The high hospitalization rates in unvaccinated compared with vaccinated persons with and without a booster dose underscores the importance of COVID-19 vaccinations in preventing hospitalizations and suggests that increasing vaccination coverage, including booster dose coverage, can prevent hospitalizations, serious illness, and death. Some of the differences in hospitalization rates between unvaccinated and vaccinated persons may be associated with differences in behavior and underlying characteristics in these groups. However, hospitalization rates were disproportionately associated with unvaccinated persons, even in early 2022, when the highly transmissible Omicron variant was the predominant variant.^8^ Although the overall hospitalization rate ratio between unvaccinated and vaccinated persons was lower during the Omicron period compared with the Delta period, hospitalization rates in those who were unvaccinated remained higher than those who were vaccinated.

Consistent with other studies, hospitalized cases among vaccinated persons occurred in older and more medically fragile populations.^23,24^ People at the greatest risk of severe disease (including those older than 75 years, with immunosuppression, with underlying medical conditions, and those who reside in LTCFs) may also be among those less likely to mount an adequate immune response to vaccination and SARS-CoV-2 infection. The study results suggest that persons with underlying conditions are more likely to be vaccinated, and those who were hospitalized despite vaccination may be more vulnerable to severe infection at baseline than those who are unvaccinated. Vaccination likely attenuates disease severity if infection occurs in a vaccinated person,^6,25^ but the current study found that conditional on being hospitalized, vaccinated persons were still at a high risk of severe outcomes. Although vaccinated patients had a shorter length of stay than unvaccinated patients, after adjusting for multiple factors, there was no clear difference in the risk for ICU admission or in-hospital death between vaccinated and unvaccinated persons, likely reflecting that those who were hospitalized despite vaccination may be more vulnerable to severe infection at baseline than those who are unvaccinated. Unidentified confounders that are not well accounted for may also be associated with these results; further detailed analyses examining clinical presentation and outcomes are ongoing.

The study finding that a substantial and growing proportion of people hospitalized with COVID-19 were vaccinated is not surprising; the proportion of hospitalized cases who are vaccinated, including those who are boosted, is expected to increase as population vaccination coverage and receipt of booster doses increases. Given high vaccination coverage, particularly in older age groups (more than 89% for those 65 years or older by April 2022 had received at least a primary vaccination series), the finding that proportionately less (75%) of hospitalized patients in that age group and that month were vaccinated is consistent with what is expected from effective vaccines. However, the high proportion of hospitalized patients who were vaccinated suggests not only a need for all people to stay up to date with vaccination, including additional boosters doses for eligible persons,^26^ but also for increased use of early outpatient antiviral treatment for patients at high risk^27^ of severe COVID-19 regardless of vaccination status^28,29,30^ and the use of preexposure prophylaxis, such as tixagevimab-cilgavimab, in patients with an immunocompromising condition that may result in an inadequate immune response to COVID-19 vaccination.^31^

Black and Hispanic patients were less likely to be vaccinated compared with White patients, potentially reflecting vaccination coverage and overall risk of infection in specific race and ethnicity groups.^32^ However, given the racial and ethnic disparities seen throughout the pandemic, the association between race and ethnicity and vaccination status among hospitalized cases should be monitored closely.^33^

### Limitations

This analysis had several limitations. Although COVID-NET covers approximately 10% of the US population, these findings may not be generalizable to the entire country. Because SARS-CoV-2 testing was conducted at the discretion of health care professionals, COVID-NET may not have captured all COVID-19–associated hospitalizations. Hospitalization rates included all patients regardless of the reason for admission, as this was not known for all patients; rates included those who were likely admitted for another reason. For analyses of sampled cases, patients admitted for reasons that were likely unrelated to COVID-19 illness were excluded. However, the reason for admission was not always clear, potentially resulting in misclassification for some cases. Even among hospitalizations for which COVID-19 was not the likely reason for admission, COVID-19 may still have been associated with clinical decisions and outcomes. In addition, misclassification of vaccination status may have occurred if there were errors in IIS data linkage.

## Conclusions

In this cross-sectional study of US adults hospitalized with COVID-19 during the first year of vaccine availability in the US, COVID-19–associated hospitalization rates in unvaccinated adults were more than 10 times higher than in vaccinated persons, a salient finding when many eligible Americans remained unvaccinated. COVID-19 vaccines, including booster doses, are strongly associated with prevention of COVID-19–associated hospitalizations, and vaccination is effective in averting serious clinical consequences. To reduce COVID-19–associated morbidity and mortality, clinicians and public health practitioners should continue to promote COVID-19 vaccinations with all recommended doses for all eligible persons.

## Notes

Supplement. eMethods. eTable 1. Categorization of Underlying Medical Conditions eTable 2. Demographics of a representative sample of hospitalized adults aged ≥18 years with laboratory-confirmed COVID-19-associated hospitalization admitted January 1, 2021–April 30, 2022, by vaccination status — COVID-NET, 13 States eTable 3. Reason for admission by demographics and vaccination status of a representative sample of hospitalized adults aged ≥18 years with laboratory-confirmed COVID-19-associated hospitalization admitted January 1, 2021–April 30, 2022 — COVID-NET, 13 States eTable 4. Month of hospital admission, code status, underlying medical conditions and time from vaccination to hospitalization among a representative sample of hospitalized adults who are unvaccinated and vaccinated with a primary series, with and without a booster dose, aged ≥18 years with laboratory-confirmed COVID-19-associated hospitalization admitted January 1, 2021–April 30, 2022 — COVID-NET, 13 States eTable 5. Multivariable model assessing factors associated with severe COVID-19 diseases (ICU, in-hospital death) in hospitalized adults with laboratory-confirmed COVID-19-associated hospitalization admitted January 1, 2021–April 30, 2022, and restricted to those with COVID-19 as a likely reason for admission — COVID-NET, 13 States eTable 6. Demographics and characteristics of propensity score matched cohort used for assessment of factors associated with severe COVID-19 diseases (ICU, in-hospital death) by vaccination status among those with COVID-19 as a likely reason for admission eTable 7. Monthly Rate ratios of COVID-19-associated hospitalizations in unvaccinated vs vaccinated (with and without a booster dose) patients among all adults aged ≥18 years and by age group, January 24, 2021–April 30, 2022 — COVID-NET, 13 States eFigure. Selection of cases for analysis of adults ≥18 years with laboratory-confirmed COVID-19-associated hospitalization admitted January 1, 2021–April 30, 2022, by vaccination status — COVID-NET, 13 States
