# Supplementary material for: COVID-19-Associated Hospitalizations Among Vaccinated and Unvaccinated Adults 18 Years or Older in 13 US States, January 2021 to April 2022
Source: JAMA Intern Med. 2022 Sep 8;182(10):1071–81. doi: 10.1001/jamainternmed.2022.4299 (PMC9459904; doi:10.1001/jamainternmed.2022.4299)
Supplement: Supplement. — eMethods. eTable 1. Categorization of Underlying Medical Conditions eTable 2. Demographics of a representative sample of hospitalized adults aged ≥18 years with laboratory-confirmed COVID-19-associated hospitalization admitted January 1, 2021–April 30, 2022, by vaccination status — COVID-NET, 13 States eTable 3. Reason for admission by demographics and vaccination status of a representative sample of hospitalized adults aged ≥18 years with laboratory-confirmed COVID-19-associated hospitalization admitted January 1, 2021–April 30, 2022 — COVID-NET, 13 States eTable 4. Month of hospital admission, code status, underlying medical conditions and time from vaccination to hospitalization among a representative sample of hospitalized adults who are unvaccinated and vaccinated with a primary series, with and without a booster dose, aged ≥18 years with laboratory-confirmed COVID-19-associated hospitalization admitted January 1, 2021–April 30, 2022 — COVID-NET, 13 States eTable 5. Multivariable model assessing factors associated with severe COVID-19 diseases (ICU, in-hospital death) in hospitalized adults with laboratory-confirmed COVID-19-associated hospitalization admitted January 1, 2021–April 30, 2022, and restricted to those with COVID-19 as a likely reason for admission — COVID-NET, 13 States eTable 6. Demographics and characteristics of propensity score matched cohort used for assessment of factors associated with severe COVID-19 diseases (ICU, in-hospital death) by vaccination status among those with COVID-19 as a likely reason for admission eTable 7. Monthly Rate ratios of COVID-19-associated hospitalizations in unvaccinated vs vaccinated (with and without a booster dose) patients among all adults aged ≥18 years and by age group, January 24, 2021–April 30, 2022 — COVID-NET, 13 States eFigure. Selection of cases for analysis of adults ≥18 years with laboratory-confirmed COVID-19-associated hospitalization admitted January 1, 2021–April 30, 2022, by vaccination status [file jamainternmed-e224299-s001.pdf]

Supplemental Online Content

Havers FP, Pham H, Taylor CA, et al. COVID-19–associated hospitalizations among vaccinated and unvaccinated adults 18 years or older in 13 US states, January 2021 to April 2022. *JAMA Intern Med*. Published online September 8, 2022. doi:10.1001/jamainternmed.2022.4299

eMethods.
eTable 1. Categorization of Underlying Medical Conditions
eTable 2. Demographics of a representative sample of hospitalized adults aged ≥18 years with laboratory-confirmed COVID-19-associated hospitalization admitted January 1, 2021–April 30, 2022, by vaccination status — COVID-NET, 13 States
eTable 3. Reason for admission by demographics and vaccination status of a representative sample of hospitalized adults aged ≥18 years with laboratory-confirmed COVID-19-associated hospitalization admitted January 1, 2021–April 30, 2022 — COVID-NET, 13 States
eTable 4. Month of hospital admission, code status, underlying medical conditions and time from vaccination to hospitalization among a representative sample of hospitalized adults who are unvaccinated and vaccinated with a primary series, with and without a booster dose, aged ≥18 years with laboratory-confirmed COVID-19-associated hospitalization admitted January 1, 2021–April 30, 2022 — COVID-NET, 13 States
eTable 5. Multivariable model assessing factors associated with severe COVID-19 disease (ICU, in-hospital death) in hospitalized adults with laboratory-confirmed COVID-19-associated hospitalization admitted January 1, 2021–April 30, 2022, and restricted to those with COVID-19 as a likely reason for admission — COVID-NET, 13 States
eTable 6. Demographics and characteristics of propensity score matched cohort used for assessment of factors associated with severe COVID-19 disease (ICU, in-hospital death) by vaccination status among those with COVID-19 as a likely reason for admission
eTable 7. Monthly Rate ratios of COVID-19-associated hospitalizations in unvaccinated vs vaccinated (with and without a booster dose) patients among all adults aged ≥18 years and by age group, January 24, 2021–April 30, 2022 — COVID-NET, 13 States
eFigure. Selection of cases for analysis of adults ≥18 years with laboratory-confirmed COVID-19-associated hospitalization admitted January 1, 2021–April 30, 2022, by vaccination status — COVID-NET, 13 States

This supplemental material has been provided by the authors to give readers additional information about their work.

© 2022 Havers FP et al. *JAMA Internal Medicine.*

Statistical supplement: COVID-19-associated hospitalizations among vaccinated and unvaccinated adults aged ≥18 years – COVID-NET, 13 states, January 1, 2021 – April 30, 2022

COVID-NET Sampling/Weighting Methodology

COVID-NET surveillance sites transmit a minimum dataset required for cases to be included in surveillance (county, age, sex, race/ethnicity, hospital admission date, and SARS-CoV-2 testing data results) on all identified cases to CDC on a weekly basis. Since the minimum data elements of site, age, and hospital admission date were known for all cases upon first report to CDC, these fields were used to stratify cases for sampling. Sampling periods were selected since sampling needed to occur on a near-real-time basis. For each sampling period, sample sizes were calculated for the entire surveillance network to achieve desired precision around clinical estimates of interest. Approaches were developed to divide the resulting necessary sample size among the 14 surveillance sites. Sites drew random samples of cases based on CDC specifications, conducted medical chart abstractions on sampled cases, and transmitted data to CDC. CDC weighted the data to reflect the sample design.

Clinical data were weighted to reflect the probability of selection and adjusted for non-response if case report forms were not completed for a given sampled case. Data were raked, as described below. This process aims to align the weighted case distribution in COVID-NET by site, age, sex, and race/ethnicity with the COVID-NET catchment population totals. Since weight adjustments yielded more dispersed weights, weights were trimmed [1] to minimize overdispersion. The full weighting process is described below:

1. *Imputation:* About 2% of sampled cases were missing race/ethnicity and 0.1% were missing sex. Both fields were used in raking procedures, so data were imputed for cases missing either field using hot deck imputation where site, age group, and admission month were used to select imputation donors.[2]
2. *Base weight*: The base weight was calculated as the total number of COVID-NET cases divided by the number of sampled cases in each sampling period, by site and age group.
3. *Non-response adjustment:* Non-response adjustment factors were calculated as the total number of sampled cases (complete and incomplete) divided by the number of completed sampled cases during each period. Factors were stratified by site and age group, and the non-

© 2022 Havers FP et al. *JAMA Internal Medicine.*

response adjusted weight was the product of the base weight and the nonresponse adjustment factor.

4. *First raking:* A SAS macro[1]was used to rake the non-response adjusted weights to known population totals.
    a. Four raking dimensions were used, listed in order below:
        1. Site by individual month
        2. Race/ethnicity by individual month
        3. Sex by individual month
        4. Age group by individual month
5. *Weight trimming:* Weight trimming was applied to the raked weights, where W_{Fi} is the final weight for the i-th case in the F-th strata, m_F is the median and σ_F is the interquartile range of the weights.
    a. $$W_{Fi} = \begin{cases} m_F + 2.9\sigma_F & \text{, if } W_{Fi} \geq m_F + 3\sigma_F \\ max(1, m_F - 3\sigma_F) & \text{, if } W_{Fi} \leq max(1, m_F - 3\sigma_F) \\ W_{Fi} & W_{Fi} \text{ , otherwise,} \end{cases}$$
6. *Second raking:* A second round of raking was applied to adjust the trimmed weights to sum to the population totals, using the same dimensions above. The final sample weight was obtained after iterating these dimensions to convergence.

Vaccination definitions, weighting of cases with known vaccination status

Vaccinated cases were defined as having received a second dose of a 2-dose series or one dose of a single-dose series ≥14 days before a positive SARS-CoV-2 test, regardless of booster dose status. If the SARS-CoV-2 test date was not available, hospital admission date was used. Patients who had received at least one dose of vaccine but had not completed a primary vaccination series were excluded from this analysis.

Vaccination status for hospitalized cases and vaccine coverage for the underlying catchment area were determined by state immunization information system (IIS) data, as previously described.[3] COVID-19 vaccination status (doses, dates administered, and product) was determined from state IISs for all sampled COVID-NET cases. In addition to the minimum data elements required for each case, some sites opted to collect vaccine information on all cases. With this additional information, non-sampled cases are able to be included in analyses regarding vaccination data. If a site did not opt to collect vaccine

information on non-sampled cases, their original sample weight is applied and only sampled cases are included in analyses. The inclusion of non-sampled cases allows COVID-NET to retain a representative sample while allowing for much more precise estimates regarding vaccine data.

Propensity Score Analysis

In the analysis comparing outcomes between vaccinated and unvaccinated hospitalized patients, the main analysis was a multivariate logistic regression model that included the entire eligible sample, and adjusted for the following covariates: race/ethnicity; age category; sex; site; long-term care facility residence; and underlying medical conditions. However, to check the robustness of our results, we also accounted for potential confounding using a second method using a propensity score matched cohort.[3,4,5] The propensity score calculated for each patient estimated the probability of vaccination use based on baseline covariates, regardless of actual vaccination status. The propensity score for each individual was calculated using a multivariable logistic regression model; the dependent variable was vaccination status. The propensity score model used included the following covariates: race/ethnicity; age category; sex; site; long-term care facility residence; and underlying medical conditions, including obesity, diabetes, chronic lung disease, cardiovascular disease, neurologic disease, renal disease, immunosuppressive conditions, liver or gastrointestinal tract disease, blood disorder, and rheumatologic or autoimmune diseases.

Using the propensity score, we matched vaccinated cases with unvaccinated cases who had similar propensity scores using a 1:1 greedy matching algorithm.[3,4,5] The propensity-matched cohort selected vaccinated and unvaccinated cases who had similar baseline characteristics except for vaccination status (Supplementary Table 4). Using these propensity-matched cohorts, models for ICU status and in-hospital death used logistic regression. To account for residual confounding, the logistic regression model adjusted for the same variables used in the model that generated the propensity score.

References:

1. Izrael D, Hoaglin DC, Battaglia MP. A SAS Macro for Balancing a Weighted Sample. 2000.

© 2022 Havers FP et al. *JAMA Internal Medicine.*

2. Wang F, Pedlow S, Wang Y. NORCSuite_Impute: Two-Way Search Hot-Deck Imputation Macro Using SAS IML. 2012 Proceedings of the American Statistical Association. Alexandria, VA2012.
3. Lori S. Parsons, Ovation Research Group, Seattle, WA. Reducing Bias in a Propensity Score Matched-Pair Sample Using Greedy Matching Techniques; https://support.sas.com/resources/papers/proceedings/proceedings/sugi26/p214-26.pdf; accessed 1/25/2022.
4. Bocheng Jing, Northern California Institute for Research and Education L. Grisell Diaz-Ramirez, University of California, San Francisco W. John Boscardin, University of California, San Francisco Michael A. Steinman, University of California, San Francisco and the San Francisco VA Medical Center. Propensity Score Matching with Survey Data. at https://www.sas.com/content/dam/SAS/support/en/sas-global-forum-proceedings/2020/4942-2020.pdf; accessed 1/23/2022.
5. Karabon P. Applying Propensity Score Methods to Complex Survey Data Using PROC PSMATCH. SAS;Paper 3634-2019. https://www.sas.com/content/dam/SAS/support/en/sas-global-forum-proceedings/2019/3634-2019.pdf; accessed February 16, 2022.

© 2022 Havers FP et al. *JAMA Internal Medicine.*

Supplementary Table 1. Categorization of Underlying Medical Conditions[a]

Chronic Lung Disease	• Active tuberculosis<br>• Asbestosis<br>• Asthma/Reactive airway disease<br>• Bronchiectasis<br>• Bronchiolitis obliterans<br>• Chronic bronchitis<br>• Chronic respiratory failure<br>• Cystic fibrosis<br>• Emphysema/Chronic Obstructive Pulmonary Disease<br>• Interstitial lung disease<br>• Obstructive sleep apnea<br>• Oxygen dependent<br>• Pulmonary fibrosis<br>• Restrictive lung disease<br>• Sarcoidosis
Diabetes Mellitus	
Other Chronic Metabolic Disease (except Diabetes Mellitus)	• Adrenal Disorders (Addison's, Adrenal Insufficiency, Cushing Syndrome, Congenital Adrenal Hyperplasia)<br>• Glycogen or other storage diseases<br>• Hyper/Hypo function of pituitary gland<br>• Inborn errors of metabolism<br>• Metabolic Syndrome<br>• Parathyroid Dysfunction (Hyperparathyroidism, Hypoparathyroidism)<br>• Thyroid dysfunction (Grave's disease, Hashimoto's Disease, Hyperthyroidism, Hypothyroidism)
Blood Disorders/Hematologic Conditions	• Alpha thalassemia<br>• Aplastic anemia<br>• Beta thalassemia<br>• Coagulopathy (Factor V Leiden, Von Willebrand Disease)<br>• Hemoglobin S-beta thalassemia<br>• Leukopenia<br>• Myelodysplastic syndrome<br>• Neutropenia<br>• Pancytopenia<br>• Polycythemia vera<br>• Sickle cell disease<br>• Splenectomy/Asplenia<br>• Thrombocytopenia
Cardiovascular Disease (except hypertension)	• Aortic aneurysm, history of<br>• Aortic/Mitral/Tricuspid/Pulmonic valve replacement, history of<br>• Aortic regurgitation<br>• Aortic stenosis<br>• Atherosclerotic cardiovascular disease<br>• Atrial fibrillation<br>• Atrioventricular blocks<br>• Automated implantable devices/Pacemaker<br>• Bundle branch block<br>• Cardiomyopathy

© 2022 Havers FP et al. *JAMA Internal Medicine.*

	• Carotid stenosis<br>• Cerebral vascular accident/Incident/Stroke, history of<br>• Congenital heart disease<br>• Coronary artery bypass grafting, history of<br>• Coronary artery disease<br>• Deep vein thrombosis, history of<br>• Heart failure/congestive heart failure<br>• Myocardial infarction, history of<br>• Mitral regurgitation<br>• Mitral stenosis<br>• Peripheral artery disease<br>• Peripheral vascular disease<br>• Pulmonary embolism, history of<br>• Pulmonary hypertension<br>• Pulmonic regurgitation<br>• Pulmonic stenosis<br>• Transient ischemic attack, history of<br>• Tricuspid regurgitation<br>• Tricuspid stenosis<br>• Ventricular fibrillation, history of<br>• Ventricular tachycardia, history of
Hypertension	
Neurologic Disorder	• Amyotrophic lateral sclerosis<br>• Cerebral palsy<br>• Cognitive dysfunction<br>• Dementia/Alzheimer's disease<br>• Developmental delay<br>• Down syndrome/Trisomy 21<br>• Edward's syndrome/Trisomy 18<br>• Epilepsy/seizure/seizure disorder<br>• Mitochondrial disorder<br>• Multiple sclerosis<br>• Muscular dystrophy<br>• Myasthenia gravis<br>• Neural tube defects/Spina bifida<br>• Neuropathy<br>• Parkinson's disease<br>• Plegias/Paralysis/Quadriplegia<br>• Scoliosis/Kyphoscoliosis<br>• Traumatic brain injury, history of
History of Guillain-Barre Syndrome	
Immunocompromised Conditions	• AIDS or CD4 count <200<br>• Complement deficiency<br>• Graft vs. host disease<br>• HIV Infection<br>• Immunoglobulin deficiency/Immunodeficiency<br>• Immunosuppressive therapy (within the last 12 months prior to admission)<br>• Leukemia[b]<br>• Lymphoma/Hodgkins/Non-Hodgkins[b]

© 2022 Havers FP et al. *JAMA Internal Medicine.*

	• Metastatic cancer[b]<br>• Multiple myeloma[b]<br>• Solid organ malignancy[b]<br>• Steroid therapy (within 2 weeks of admission)<br>• Transplant, hematopoietic stem cell (Bone marrow transplant, peripheral stem cell transplant), history of<br>• Transplant, solid organ, history of
Renal Disease	• Chronic kidney disease/chronic renal insufficiency<br>• Dialysis<br>• End stage renal disease<br>• Glomerulonephritis<br>• Nephrotic syndrome<br>• Polycystic kidney disease
Gastrointestinal/Liver Disease	• Alcoholic hepatitis<br>• Autoimmune hepatitis<br>• Barrett's esophagitis<br>• Chronic liver disease<br>• Chronic pancreatitis<br>• Cirrhosis/End stage liver disease<br>• Crohn's disease<br>• Esophageal varices<br>• Esophageal strictures<br>• Hepatitis B, chronic<br>• Hepatitis C, chronic<br>• Non-alcoholic fatty liver disease/Non-alcoholic steatohepatitis<br>• Ulcerative colitis
Rheumatologic/Autoimmune Conditions	• Ankylosing spondylitis<br>• Dermatomyositis<br>• Juvenile idiopathic arthritis<br>• Kawasaki disease<br>• Microscopic polyangiitis<br>• Polyarteritis nodosum<br>• Polymyalgia rheumatica<br>• Polymyositis<br>• Psoriatic arthritis<br>• Rheumatoid arthritis<br>• Systemic Lupus Erythematosus/Lupus<br>• Systemic sclerosis<br>• Takayasu arteritis<br>• Temporal/Giant Cell arteritis<br>• Vasculitis, other
Other, specify	• Post-partum (2 weeks or less)<br>• Feeding tube dependent<br>• Tracheostomy dependent/ventilator dependent<br>• Wheelchair dependent<br>• Free text field for other underlying medical conditions that were not specified above

[a]Underlying medical conditions were selected before hospitalization surveillance began.
[b]Current/in treatment or diagnosed in previous 12 months

© 2022 Havers FP et al. *JAMA Internal Medicine.*

Supplementary Table 2. Demographics of a representative sample of hospitalized adults aged ≥18 years with laboratory-confirmed COVID-19-associated hospitalization admitted January 1, 2021–April 30, 2022, by vaccination status — COVID-NET, 13 States[a]

Characteristics	Adults ≥18 years	Unvaccinated n (weighted %) [b]	Partially vaccinated, n (weighted %) [b,c]	Vaccinated with a primary series without a booster dose n (weighted %) [b]	Vaccinated with a booster dose [b]
**Total[d]**	**146,658**	**98,244 (69.3)**	**9,062 (6.2)**	**30,556 (19.1)**	**8,796 (5.4)**
**Age group** (median, IQR)	60 (45-73)	57 (42-69)	65 (53-77)	67 (53-78)	72 (59-81)
18–49 years	44,716	35,354 (80.7)	2,116 (4.3)	6,022 (12.4)	1,224 (2.6)
50–64 years	40,115	28,636 (74.2)	2,537 (5.9)	7,257 (16.2)	1,685 (3.7)
65–74 years	27,841	17,149 (63.3)	1,810 (7.1)	6,819 (23.2)	2,063 (6.4)
75–84 years	21,269	11,144 (54.9)	1,568 (7.3)	6,353 (28.1)	2,204 (9.7)
≥85 years	12,717	5,961 (50.6)	1,031 (9.8)	4,105 (28.3)	1,620 (11.2)
**Sex**					
Male	72,443	48,561 (69.4)	4,511 (6.3)	14,987 (18.9)	4,384 (5.3)
Female	74,215	49,683 (69.2)	4,551 (6.0)	15,569 (19.4)	4,412 (5.4)
**Race and Ethnicity[e]**					
White	73,135	45,888 (64.9)	4,504 (6.1)	17,194 (22.0)	5,549 (7.0)
Black	33,584	24,166 (76.3)	2,288 (6.4)	5,972 (14.7)	1,158 (2.6)
Hispanic or Latino	21,937	16,174 (74.5)	1,216 (6.0)	3,660 (15.8)	887 (3.8)
American Indian or Alaska Native	1,941	1,363 (66.7)	150 (8.7)	338 (20.4)	90 (4.2)
Asian or Pacific Islander	5,837	3,632 (65.2)	392 (7.4)	1,295 (20.4)	518 (6.9)
Other/Unknown[f]	10,027	6,884 (69.6)	504 (4.9)	2,061 (20.0)	578 (5.5)
**Month of admission**					
January 2021	11,146	10,580 (93.9)	564 (6.1)	2 (0.0)	0 (0.0)
February 2021	7,859	6,935 (88.9)	863 (10.4)	61 (0.7)	0 (0.0)
March 2021	6,666	5,563 (85.3)	895 (11.6)	208 (3.1)	0 (0.0)
April 2021	8,029	6,483 (81.3)	1,144 (13.4)	402 (5.3)	0 (0.0)
May 2021	5,237	4,269 (80.9)	480 (9.7)	488 (9.4)	0 (0.0)

© 2022 Havers FP et al. *JAMA Internal Medicine.*

June 2021	2,307	1,800 (76.6)	143 (7.0)	364 (16.3)	0 (0.0)
July 2021	4,481	3,461 (77.2)	209 (4.4)	811 (18.4)	0 (0.0)
August 2021	12,101	9,159 (74.2)	654 (4.8)	2,288 (20.9)	0 (0.0)
September 2021	10,688	7,727 (72.0)	535 (4.0)	2,400 (23.4)	26 (0.6)
October 2021	10,258	6,795 (66.2)	442 (4.1)	2,950 (29.1)	71 (0.6)
November 2021	10,506	6,952 (65.6)	394 (3.4)	2,963 (28.7)	197 (2.3)
December 2021	14,686	9,077 (63.6)	626 (4.0)	4,203 (27.6)	780 (4.8)
January 2022	27,723	13,693 (47.7)	1,415 (5.2)	9,097 (33.5)	3,518 (13.6)
February 2022	8,721	3,733 (42.5)	434 (4.7)	2,621 (30.8)	1,933 (22.0)
March 2022	2,932	1,063 (35.3)	139 (4.8)	799 (26.7)	931 (33.3)
April 2022	3,318	954 (29.7)	125 (3.3)	899 (25.9)	1,340 (41.1)

Abbreviations: COVID-19 = coronavirus disease 2019; COVID-NET = COVID-19–Associated Hospitalization Surveillance Network; IQR = interquartile range.
[a]California, Connecticut, Colorado, Georgia, Maryland (data excluded beginning December 4, 2021), Michigan, Minnesota, New Mexico, New York, Ohio, Oregon, Tennessee, and Utah
[b]Percentages are weighted and represent row percentages
[c] 8,153 (6.3%) of hospitalized cases were partially vaccinated at the time of hospitalization and excluded from further analyses.
[d]318 additional cases were excluded from all analyses due to unknown vaccination status
[e]Data on race and ethnicity were categorized as follows: non-Hispanic White (White), non-Hispanic Black (Black), non-Hispanic Asian or Pacific Islander (Asian/Pacific Islander), non-Hispanic American Indian or Alaska Native (American Indian/Alaska Native) and Other/Unknown. If ethnicity was unknown (8% of cases), non-Hispanic ethnicity was assumed.
[f]Includes multiple race (615, 0.4%) and unknown race (9,412, 5%).

© 2022 Havers FP et al. *JAMA Internal Medicine.*

Supplementary Table 3. Reason for admission by demographics and vaccination status of a representative sample of hospitalized adults aged ≥18 years with laboratory-confirmed COVID-19-associated hospitalization admitted January 1, 2021–April 30, 2022 — COVID-NET, 13 States[a]

Characteristics	Total	COVID-19 related illness [b]	OB/Labor and delivery admission [b]	Surgery [b]	Psychiatric admission needing acute medical care [b]	Trauma [b]	Other [b]	Unknown reason [b]
**Total**	**13,043**	**11,127 (86.6)**	**707 (5.0)**	**219 (1.4)**	**270 (2.2)**	**290 (2.0)**	**190 (1.7)**	**240 (1.2)**
Age group								
18-49 years	4,477	3,220 (71.9)	707 (16.3)	77 (1.5)	182 (4.6)	118 (2.3)	102 (2.4)	71 (1.0)
50-64 years	4,328	3,988 (92.6)	0 (0.0)	82 (1.6)	61 (1.4)	62 (1.6)	49 (1.5)	86 (1.3)
65+ years	4,238	3,919 (93.5)	0 (0.0)	60 (1.1)	27 (0.9)	110 (2.1)	39 (1.2)	83 (1.2)
65-74 years	1,949	1,806 (94.1)	0 (0.0)	33 (0.9)	16 (0.8)	34 (1.6)	20 (1.2)	40 (1.3)
75-84 years	1,466	1,363 (94.5)	0 (0.0)	16 (1.2)	6 (0.4)	41 (1.6)	12 (1.3)	28 (1.0)
85+ years	823	750 (90.7)	0 (0.0)	11 (1.3)	5 (1.7)	35 (3.8)	7 (1.2)	15 (1.3)
**Sex**								
Male	6,433	5,759 (90.8)	0 (0.0)	115 (1.2)	151 (2.6)	174 (2.4)	102 (1.9)	132 (1.3)
Female	6,610	5,368 (82.6)	707 (9.8)	104 (1.6)	119 (1.8)	116 (1.6)	88 (1.5)	108 (1.1)
**Race/Ethnicity[c]**								
White	7,305	6,342 (88.9)	260 (3.0)	111 (1.1)	148 (1.8)	169 (2.1)	107 (1.6)	168 (1.5)
Black	2,628	2,230 (85.8)	172 (6.1)	43 (1.0)	58 (2.4)	57 (2.2)	32 (1.6)	36 (0.9)
American Indian or Alaska Native	184	160 (88.0)	10 (7.4)	1 (0.4)	4 (1.6)	4 (1.1)	4 (1.1)	1 (0.3)
Asian or Pacific Islander	515	429 (81.2)	46 (7.9)	10 (1.7)	11 (4.9)	9 (0.8)	9 (3.0)	1 (0.6)
Hispanic or Latino	1,877	1,531 (82.6)	185 (9.2)	46 (2.9)	32 (1.2)	35 (1.5)	34 (2.3)	14 (0.4)
Other/Unknown[d]	533	435 (83.9)	34 (4.3)	8 (1.8)	17 (4.8)	16 (3.1)	4 (0.3)	19 (1.9)
**Vaccination Status**								
Unvaccinated	9,994	8,575 (87.5)	582 (5.3)	136 (1.0)	202 (2.0)	191 (1.9)	116 (1.2)	192 (1.2)
Vaccinated (with or without a booster dose)	3,049	2,552 (84.3)	125 (4.2)	83 (2.3)	68 (2.7)	99 (2.3)	74 (3.1)	48 (1.1)
Vaccinated without a booster dose	2,432	2,061 (85.4)	86 (4.3)	54 (1.9)	52 (2.4)	84 (2.2)	59 (2.8)	36 (1.0)
Boosted	617	491 (80.5)	39 (4.0)	29 (3.8)	16 (3.7)	15 (2.4)	15 (4.1)	12 (1.5)
**Time period**								
Pre-Delta (January 2020 – June 2021)	5,657	4,819 (86.9)	327 (5.0)	118 (1.8)	117 (2.0)	135 (2.2)	67 (1.2)	74 (0.9)
Delta (July 2021 – December 2021)	4,882	4,369 (91.9)	163 (2.9)	35 (0.4)	69 (1.4)	7.1 (2.2)	64 (1.2)	111 (1.3)
Omicron (January 2022 – April 2022)	2,504	1,939 (80.3)	217 (7.4)	66 (2.0)	84 (3.3)	84 (2.7)	59 (2.9)	55 (1.3)

Abbreviations: COVID-19 = coronavirus disease 2019; COVID-NET = COVID-19–Associated Hospitalization Surveillance Network; IQR = interquartile range.

[a] California, Connecticut, Colorado, Georgia, Maryland (data excluded beginning December 4, 2021), Michigan, Minnesota, New Mexico, New York, Ohio, Oregon, Tennessee, and Utah.

© 2022 Havers FP et al. *JAMA Internal Medicine.*

[b] Percentages are weighted and represent row percentages
[c]Data on race and ethnicity were categorized as follows: non-Hispanic White (White), non-Hispanic Black (Black), non-Hispanic Asian or Pacific Islander (Asian/Pacific Islander), non-Hispanic American Indian or Alaska Native (American Indian/Alaska Native) and Other/Unknown. If ethnicity was unknown (8% of cases), non-Hispanic ethnicity was assumed.
[d]Includes multiple race (71, 0.7%) and unknown race (462, 4%).

© 2022 Havers FP et al. *JAMA Internal Medicine.*

Supplementary Table 4. Month of hospital admission, code status, underlying medical conditions and time from vaccination to hospitalization among a representative sample of hospitalized adults who are unvaccinated and vaccinated with a primary series, with and without a booster dose, aged ≥18 years with laboratory-confirmed COVID-19-associated hospitalization admitted January 1, 2021–April 30, 2022 — COVID-NET, 13 States[a]

Category	Adults ≥18 years (January 2021 – April 2022)				Vaccinated adults ≥18 years (October 2021-April 2022)[b]			
	Total	Unvaccinated n (weighted %) [b]	Vaccinated with a primary series with and without a booster dose, n (weighted %) [b]	p-value[d]	Total	Vaccinated without booster, n (weighted %)	Vaccinated with booster, n (weighted %)	p-value[c]
**Month of hospital admission (row percent)**				**<.001**				**<.001**
January 2021	853	853 (100.0)	0 (0.0)					
February 2021	741	738 (99.7)	3 (0.3)					
March 2021	740	718 (96.4)	22 (3.6)					
April 2021	751	701 (93.4)	50 (6.6)					
May 2021	982	867 (89.7)	115 (10.3)					
June 2021	832	668 (82.6)	164 (17.4)					
July 2021	785	597 (80.1)	188 (19.9)					
August 2021	817	615 (77.8)	202 (22.2)					
September 2021	1,011	753 (74.6)	258 (25.4)					
October 2021	706	489 (68.4)	217 (31.6)		217	212 (98.3)	5 (1.7)	
November 2021	685	494 (67.1)	191 (32.9)		191	177 (91.8)	14 (8.2)	
December 2021	553	380 (68.3)	173 (31.7)		173	144 (83.8)	29 (16.2)	
January 2022	491	250 (49.0)	241 (51.0)		241	171 (69.8)	70 (30.2)	
February 2022	394	172 (47.0)	222 (53.0)		222	141 (62.8)	81 (37.2)	
March 2022	405	157 (35.9)	248 (64.1)		248	119 (48.3)	129 (51.7)	
April 2022	381	123 (25.7)	258 (74.3)		258	99 (29.6)	159 (70.4)	

© 2022 Havers FP et al. *JAMA Internal Medicine.*

Category	Adults ≥18 years (January 2021 – April 2022)				Vaccinated adults ≥18 years (October 2021-April 2022)[b]			
	Total	Unvaccinated n (weighted %) [b]	Vaccinated with a primary series with and without a booster dose, n (weighted %) [b]	p-value[d]	Total	Vaccinated without booster, n (weighted %)	Vaccinated with booster, n (weighted %)	p-value[c]
**Code status on admission**				**<.001**				0.486
Full code	9,348	7,323 (84.9)	2,025 (78.6)		1,237	867 (79.2)	370 (75.7)	
DNR/DNI/CMO	830	489 (6.6)	341 (14.7)		197	122 (14.3)	75 (17.6)	
Unknown	949	763 (8.5)	186 (6.7)		116	74 (6.5)	42 (6.7)	
**Underlying medical condition**[d]								
**Any underlying medical condition**	10,116	7,658 (89.9)	2,458 (96.9)	**<.001**	1,487	1,016 (97.4)	471 (95.8)	0.280
**Hypertension**	6,012	4,252 (53.5)	1,760 (71.6)	**<.001**	1,039	693 (71.4)	346 (71.5)	0.982
**Obesity**[e]	5,368	4,330 (47.9)	1,038 (38.0)	**<.001**	613	441 (37.9)	172 (31.1)	0.096
**Chronic metabolic disease**	4,392	3,096 (38.8)	1,296 (51.9)	**<.001**	774	530 (52.9)	244 (47.2)	0.176
**Diabetes mellitus**	3,448	2,455 (31.4)	993 (40.2)	**<.001**	588	405 (40.5)	183 (35.9)	0.267
**Chronic lung disease**	5,734	3,953 (48.2)	1,781 (70.0)	**<.001**	1,078	728 (70.2)	350 (69.7)	0.906
Asthma/Reactive airway disease	1,546	1,149 (12.6)	397 (14.0)	0.196	248	172 (14.4)	76 (13.3)	0.657
COPD	1,171	730 (9.3)	441 (19.4)	**<.001**	285	190 (19.9)	95 (21.0)	0.756
**Cardiovascular disease**[f]	3,744	2,379 (31.6)	1,365 (53.9)	**<.001**	827	542 (53.3)	285 (56.7)	0.423
Heart failure	1,229	705 (10.8)	524 (20.1)	**<.001**	310	199 (19.1)	111 (23.7)	0.163
Coronary artery disease	1,340	842 (11.3)	498 (18.7)	**<.001**	297	192 (17.1)	105 (24.2)	**0.033**
**Neurologic disease**	1,883	1,157 (14.8)	726 (30.2)	**<.001**	429	286 (31.5)	143 (30.3)	0.776
**Renal disease**	1679	992 (14.4)	687 (30.1)	**<.001**	398	245 (29.9)	153 (34.2)	0.299
**Immunosuppressive condition**	1,437	877 (10.8)	560 (23.3)	**<.001**	342	198 (19.9)	144 (32.5)	**0.001**
Solid organ malignancy	511	301 (3.7)	210 (9.6)	**<.001**	122	77 (9.2)	45 (9.7)	0.838
Transplant	170	81 (1.5)	89 (4.4)	**<.001**	54	21 (2.5)	33 (10.4)	0.838

© 2022 Havers FP et al. *JAMA Internal Medicine.*

Category	Adults ≥18 years (January 2021 – April 2022)				Vaccinated adults ≥18 years (October 2021-April 2022)[b]			
	Total	Unvaccinated n (weighted %) [b]	Vaccinated with a primary series with and without a booster dose, n (weighted %) [b]	p-value[d]	Total	Vaccinated without booster, n (weighted %)	Vaccinated with booster, n (weighted %)	p-value[c]
**Gastrointestinal or liver disease**	1,150	81 (1.5)	355 (12.9)	**0.002**	211	149 (13.8)	62 (10.4)	0.259
**Blood disorder**	439	269 (3.0)	170 (7.0)	**<.001**	102	64 (8.2)	38 (6.0)	0.318
**Rheumatologic or autoimmune disease**	774	465 (5.9)	309 (13.5)	**<.001**	184	107 (12.3)	77 (19.1)	0.0327
**Vaccination to Admission time,[g] Median (IQR)**			180 (103-246)		211 (109-262)			
14-90 days after vaccination			556 (19.0)		207	42 (5.7)	165 (44.7)	
91-150 days after vaccination			584 (18.2)		274	76 (7.0)	198 (39.7)	
>=150 days after vaccination			1,372 (61.4)		1,043	932 (86.1)	111 (13.3)	

Abbreviations: Abbreviations: COVID-19 = coronavirus disease 2019; COVID-NET = COVID-19–Associated Hospitalization Surveillance Network; DNI = do not intubate; DNR = do not resuscitate; CMO=comfort measure only; COPD=Chronic obstructive pulmonary disease; ICU = intensive care unit; IQR = interquartile range;

[a]California, Connecticut, Colorado, Georgia, Maryland (data excluded beginning December 4, 2021), Michigan, Minnesota, New Mexico, New York, Ohio, Oregon, Tennessee, and Utah. Note that column percentages are shown except where row percentages are indicated.

[b] **Unvaccinated:** Population-based rates of COVID-19-associated hospitalizations among persons with a positive SARS-CoV-2 test who have no record of receiving any COVID-19 vaccine; **Vaccinated:** Population-based rates of COVID-19-associated hospitalizations among persons with a positive SARS-CoV-2 test collected ≥14 days after vaccination with a primary series, defined as either the second dose of a two-dose vaccine series or after one dose of a single dose vaccine. When not otherwise specified, vaccinated persons include those who may have received additional or booster doses; **Vaccinated without a booster dose:** Population-based rates of COVID-19-associated hospitalizations among vaccinated persons who have received a primary series and who have not received an additional or booster dose. This includes both those eligible and not yet eligible for an additional or booster dose; **Vaccinated with a booster dose:** Population-based rates of COVID-19-associated hospitalizations among persons vaccinated with a primary series who have received an additional or booster dose on or after August 13, 2021, with a positive SARS-CoV-2 test collected ≥14 days after receipt of an additional or booster dose. Because the immune status of all cases is not known, an additional dose (recommended for persons with a weakened immune system) cannot be distinguished from a booster dose. This is a relevant consideration because vaccines can be less effective in persons with a weakened immune system.

[c] Statistical significance for univariate analyses was determined as p<0.10

[d] Overall condition categories as defined in Supplementary Table 1.

[e]Obesity is defined as calculated body mass index (BMI) ≥30 kg/m2, and if BMI is missing, by International Classification of Diseases discharge diagnosis codes.

[f] Cardiovascular disease excludes hypertension.

[g] Time from vaccination to admission is the number of days between the most last vaccine dose received plus 14 days and date of hospital admission.

© 2022 Havers FP et al. *JAMA Internal Medicine.*

Supplementary Table 5. Multivariable model[a] assessing factors associated with severe COVID-19 disease (ICU, in-hospital death) in hospitalized adults with laboratory-confirmed COVID-19-associated hospitalization admitted January 1, 2021–April 30, 2022, and restricted to those with COVID-19 as a likely reason for admission — COVID-NET, 13 States[b]

Category	No severe illness n (weighted %)	Have severe illness n (weighted %)	Unadjusted risk ratio	Adjusted risk ratio	p-value
**Age group**	61 (48-74)	63 (52-74)			
18-49 years	2,590 (27.1)	630 (20.2)	Ref.	Ref.	Ref.
50-64 years	2,975 (29.2)	1,013 (31.9)	1.35 (1.2, 1.51)	1.33 (1.19, 1.47)	**<.001**
65+ years	2,907 (43.7)	1,012 (47.9)	1.35 (1.21, 1.51)	1.34 (1.19, 1.5)	**<.001**
**Sex**					
Male	4,218 (50.2)	1,541 (56.6)	1.22 (1.12, 1.32)	1.23 (1.14, 1.33)	**<.001**
Female	4,254 (49.8)	1,114 (43.4)	Ref.	Ref.	Ref.
**Race/Ethnicity[c]**					
White	4,853 (51.1)	1,489 (52.5)	Ref.	Ref.	Ref.
Black	1,705 (25.5)	525 (22.9)	0.9 (0.8, 1.02)	0.98 (0.84, 1.15)	0.850
Hispanic or Latino	1,170 (12.5)	361 (12.8)	1 (0.9, 1.11)	1.07 (0.95, 1.2)	0.281
American Indian or Alaska Native	103 (1.2)	57 (1.7)	1.27 (1.03, 1.56)	1.3 (1.06, 1.6)	**0.013**
Asian or Pacific Islander	313 (4.7)	116 (4.7)	0.98 (0.78, 1.23)	1.06 (0.86, 1.31)	0.590
Other/Unknown[d]	328 (4.9)	107 (5.4)	1.05 (0.85, 1.3)	1.11 (0.93, 1.34)	0.256
**Time period**					
Pre-Delta (January 2021- June 2021)	3,737 (35.6)	1,082 (32.1)	Ref.	Ref.	Ref.
Delta (July 2021- December 2021)	3,193 (35.7)	1,176 (43.5)	1.26 (1.12, 1.41)	1.34 (1.16, 1.55)	**<.001**
Omicron (January 2022- April 2022)	1,542 (28.8)	397 (24.5)	0.96 (0.82, 1.11)	1.01 (0.82, 1.24)	0.911
**Vaccinated with a primary series with or without a booster dose**	1,984 (25.6)	568 (24.7)	0.96 (0.8, 1.16)	0.83 (0.65, 1.07)	0.158
**LTCF residence[e]**	425 (6.4)	179 (8.2)	1.22 (0.99, 1.51)	1.24 (0.98, 1.56)	0.070
**Underlying medical condition**					
Obesity[f]	4,054 (44.9)	1,314 (47.0)	1.07 (0.96, 1.19)	1.1 (0.98, 1.23)	0.097
Diabetes	2,474 (31.9)	974 (39.2)	1.27 (1.18, 1.38)	1.22 (1.13, 1.32)	**<.001**
Chronic lung disease	4,297 (53.0)	1,437 (56.2)	1.1 (1.02, 1.19)	1.02 (0.94, 1.11)	0.621
Cardiovascular disease[g]	2,756 (36.4)	988 (40.2)	1.13 (1.02, 1.25)	1.01 (0.9, 1.14)	0.805
Neurologic disease	1,404 (18.5)	479 (19.4)	1.05 (0.91, 1.2)	1 (0.84, 1.17)	0.960
Renal disease	1,198 (17.5)	481 (21.1)	1.19 (1.05, 1.35)	1.11 (0.99, 1.25)	0.066
Immunosuppressive condition	1,057 (13.4)	380 (16.1)	1.18 (1.04, 1.34)	1.17 (1, 1.38)	0.056
Gastrointestinal or liver disease	843 (10.0)	307 (10.9)	1.08 (0.89, 1.31)	1.03 (0.86, 1.23)	0.754
Blood disorder	312 (4.0)	127 (4.1)	1.02 (0.76, 1.36)	0.96 (0.73, 1.28)	0.795

© 2022 Havers FP et al. *JAMA Internal Medicine.*

Rheumatologic or autoimmune disease	572 (7.4)	202 (9.1)	1.19 (0.88, 1.59)	1.09 (0.77, 1.53)	0.628

Abbreviations: COVID-19 = coronavirus disease 2019; COVID-NET = COVID-19–Associated Hospitalization Surveillance Network; LTCF = long-term care facility

[a]Log-linked Poisson Regression using generalized estimating equations (GEE) clustered on site with exchangeable covariance structure

[b]California, Connecticut, Colorado, Georgia, Maryland (data excluded beginning December 4, 2021), Michigan, Minnesota, New Mexico, New York, Ohio, Oregon, Tennessee, and Utah.

[c]Data on race and ethnicity were categorized as follows: non-Hispanic White (White), non-Hispanic Black (Black), Non-Hispanic American Indian or Alaska Native, Non-Hispanic Asian or Pacific Islander, and, Other/Unknown. If ethnicity was unknown (8%), non-Hispanic ethnicity was assumed.

[d]Includes multiple race (53, .5%) and unknown race (382, 4.5%)

[e]Long-term care facility (LTCF) residence was defined as residence in rehabilitation facilities, assisted living/residential care, group homes, nursing homes, skilled nursing facilities, long-term care facilities, long-term acute care hospitals, residential care facilities or other long-term care facilities.

[f]Obesity is defined as calculated body mass index (BMI) ≥30 kg/m2, and if BMI is missing, by International Classification of Diseases discharge diagnosis codes.

[g]Cardiovascular disease excludes hypertension.

© 2022 Havers FP et al. *JAMA Internal Medicine.*

Supplementary Table 6. Demographics and characteristics of propensity score matched cohort used for assessment of factors associated with severe COVID-19 disease (ICU, in-hospital death) by vaccination status among those with COVID-19 as a likely reason for admission

Category	Total	Unvaccinated, n (weighted %)	Vaccinated[a], n (weighted %)	P-value
**Total**	**4,000**	**2,000 (55.0)**	**2,000 (45.0)**	
**Age group (Median, IQR)**				0.594
18-49 years	698	353 (15.1)	345 (16.2)	
50-64 years	1,303	656 (25.4)	647 (26.7)	
65+ years	1,999	991 (59.5)	1,008 (57.1)	
**Sex**				0.579
Male	2,054	1,043 (51.3)	1,011 (52.7)	
Female	1,946	957 (48.7)	989 (47.3)	
**Race/Ethnicity[b]**				0.879
White	2,634	1,316 (58.4)	1,318 (57.2)	
Black	633	316 (21.4)	317 (19.9)	
Hispanic or Latino	413	207 (9.4)	206 (11.2)	
American Indian or Alaska Native	55	28 (1.5)	27 (1.7)	
Asian or Pacific Islander	128	65 (4.3)	63 (4.3)	
Other/Unknown[c]	137	68 (5.1)	69 (5.7)	
**Time period (row percent)**				**0.005**
Pre-Delta (January 2021 - June 2021)	609	276 (10.6)	333 (6.5)	
Delta (July 2021 - December 2021)	2,025	1,072 (42.4)	953 (44.8)	
Omicron (January 2022 - April 2022)	1,366	652 (47.0)	714 (48.8)	
**LTCF residence[d]**	309	125 (7.6)	184 (7.3)	0.846
**Obesity[e]**	1,669	852 (38.7)	817 (39.1)	0.873
**Diabetes**	1,387	677 (36.2)	710 (37.8)	0.508
**Chronic lung disease**	2,565	1,276 (63.9)	1,289 (63.6)	0.914
**Cardiovascular disease[f]**	1,873	920 (48.2)	953 (47.1)	0.675
**Neurologic disease**	889	419 (21.7)	470 (23.7)	0.350
**Renal disease**	818	387 (22.9)	431 (22.6)	0.895
**Immunosuppressive condition**	717	360 (16.9)	357 (19.0)	0.280
**Gastrointestinal or liver disease**	492	226 (11.5)	266 (12.7)	0.473

© 2022 Havers FP et al. *JAMA Internal Medicine.*

**Blood disorder**	207	99 (5.0)	108 (5.0)	0.959
**Rheumatologic or autoimmune disease**	382	196 (9.0)	186 (10.7)	0.286

Abbreviations: COVID-19 = coronavirus disease 2019; COVID-NET = COVID-19–Associated Hospitalization Surveillance Network; ICU = intensive care unit.

[a] Vaccinated persons include those who were vaccinated with a primary series and who may have received additional or booster doses

[b]Data on race and ethnicity were categorized as follows: Hispanic or Latino, non-Hispanic White (White), non-Hispanic Black (Black), Non-Hispanic American Indian or Alaska Native, Non-Hispanic Asian or Pacific Islander, and Other/Unknown. If ethnicity was unknown (8% of cases), non-Hispanic ethnicity was assumed.

[c]Includes multiple race (20, 0.6%) and unknown race (117, 4.8%)

[d]Long-term care facility (LTCF) residence was defined as residence in rehabilitation facilities, assisted living/residential care, group homes, nursing homes, skilled nursing facilities, long-term care facilities, long-term acute care hospitals, residential care facilities or other long-term care facilities.

[e]Obesity is defined as calculated body mass index (BMI) ≥30 kg/m2, and if BMI is missing, by International Classification of Diseases discharge diagnosis codes.

[f]Cardiovascular disease excludes hypertension.

© 2022 Havers FP et al. *JAMA Internal Medicine.*

Supplementary Table 7. Monthly rate ratios of COVID-19-associated hospitalizations in unvaccinated vs vaccinated (with and without a booster dose), [a] patients among all adults aged ≥18 years and by age group, [b] January 24, 2021–April 30, 2022 — COVID-NET, 13 States[c]

	Rate ratio vaccinated with a primary series with or without a booster vs. unvaccinated				Rate ratio vaccinated with a primary series without booster vs. unvaccinated				Rate ratio vaccinated with a primary series with booster vs. unvaccinated				Rate ratio vaccinated with a primary series and booster vs. vaccinated with primary series without booster			
	all adults ≥18 years (age-adjusted)	18–49 years	50–64 years	≥65 years	all adults ≥18 years (age-adjusted)	18–49 years	50–64 years	≥65 years	all adults ≥18 years (age-adjusted)	18–49 years	50–64 years	≥65 years	all adults ≥18 years (age-adjusted)	18–49 years	50–64 years	≥65 years
**2021**					—	—	—	—	—	—	—	—	—	—	—	—
February	5.2	15.6	12.9	3.0	—	—	—	—	—	—	—	—	—	—	—	—
March	7.9	17.0	8.2	6.0	—	—	—	—	—	—	—	—	—	—	—	—
April	16.3	27.5	25.7	11.3	—	—	—	—	—	—	—	—	—	—	—	—
May	17.7	19.0	22.1	15.2	—	—	—	—	—	—	—	—	—	—	—	—
June	12.9	13.8	17.1	10.6	—	—	—	—	—	—	—	—	—	—	—	—
July	13.4	20.2	18.6	9.0	—	—	—	—	—	—	—	—	—	—	—	—
August	13.2	20.7	20.4	8.8	—	—	—	—	—	—	—	—	—	—	—	—
September	13.6	24.0	19.8	9.5	13.8	24.6	19.9	9.7	5.6	3.7	11.6	5.2	0.4	0.1	0.6	0.5
October	11.8	15.7	14.3	9.9	11.2	15.8	14.1	9.1	23.1	12.8	23.5	33.5	2.1	0.8	1.7	3.7
November	13.2	16.1	15.6	11.6	10.5	15.7	14.3	8.4	37.8	27.5	47.5	38.4	3.6	1.7	3.3	4.6
December	12.5	9.4	12.8	13.7	8.1	8.3	10.2	7.4	32.6	24.2	29.9	38.4	4.0	2.9	2.9	5.2
**2022**																
January	7.1	4.7	6.1	8.8	4.4	4.0	4.3	4.5	12.8	7.9	11.2	16.5	2.9	2.0	1.7	3.6
February	6.2	4.1	5.4	7.7	4.2	3.5	3.9	4.5	8.8	5.7	8.0	10.8	2.1	1.6	2.1	2.4
March	4.6	3.3	4.4	5.3	3.8	3.8	4.0	3.8	4.9	2.8	4.8	6.4	1.3	0.8	1.2	1.7
April	3.5	3.0	3.5	3.7	3.3	3.2	3.1	3.4	3.6	2.9	3.9	3.9	1.1	0.9	1.3	1.2
**Range**	3.5-17.7	3.0-27.5	3.5-25.7	3.0-15.2	3.3-13.8	3.2-24.6	3.1-19.9	3.4-9.7	3.6-37.8	2.8-27.5	3.9-47.5	3.9-38.4	0.4-4.0	0.1-2.9	0.6-3.3	0.5-5.2
**January-June 2021 Cumulative rate ratio per 100,000 person-weeks (95% CI)**	**14.9 (14.2-15.5)**	**19.7 (17.4-22.3)**	**21.3 (19.2-23.6)**	**11.1 (10.6-11.7)**	—	—	—	—	—	—	—	—	—	—	—	—

© 2022 Havers FP et al. *JAMA Internal Medicine.*

**July-December 2021 Cumulative rate ratio per 100,000 person-weeks (95% CI)**	**12.2 (12.0-12.4)**	**14.8 (14.2-15.3)**	**15.4 (15.0-16.0)**	**10.1 (9.8-10.3)**	**—**	**—**	**—**	**—**	**—**	**—**	**—**	**—**	**—**	**—**	**—**	**—**
**January-April 2022 Cumulative rate ratio per 100,000 person-weeks (95% CII)**	**6.8 (6.6-6.9)**	**4.6 (4.4-4.8)**	**5.9 (5.6-6.1)**	**8.3 (8.1-8.5)**	**—**	**—**	**—**	**—**	**—**	**—**	**—**	**—**	**—**	**—**	**—**	**—**

[a] **Unvaccinated:** Population-based rates of COVID-19-associated hospitalizations among persons with a positive SARS-CoV-2 test who have no record of receiving any COVID-19 vaccine; **Vaccinated:** Population-based rates of COVID-19-associated hospitalizations among persons with a positive SARS-CoV-2 test collected ≥14 days after vaccination with a primary series, defined as either the second dose of a two-dose vaccine series or after one dose of a single dose vaccine. When not otherwise specified, vaccinated persons include those who may have received additional or booster doses; **Vaccinated no booster:** Population-based rates of COVID-19-associated hospitalizations among vaccinated persons who have received a primary series and who have not received an additional or booster dose. This includes both those eligible and not yet eligible for an additional or booster dose; **Vaccinated with booster dose:** Population-based rates of COVID-19-associated hospitalizations among persons vaccinated with a primary series who have received an additional or booster dose on or after August 13, 2021, with a positive SARS-CoV-2 test collected ≥14 days after receipt of an additional or booster dose. Because the immune status of all cases is not known, an additional dose (recommended for persons with a weakened immune system) cannot be distinguished from a booster dose. This is a relevant consideration because vaccines can be less effective in persons with a weakened immune system.
[b]Boxes shaded in grey indicate months where age-specific rate ratios are likely not stable, as less than 5% of the age group-specific population of the COVID-NET surveillance catchment area had received additional or booster doses 14 or more days prior to the timeframe indicated. Cells represented by a dash (—) are time periods where either booster doses were not available or very few individuals had received a booster dose.
[c]California, Connecticut, Colorado, Georgia, Maryland (data excluded beginning December 4, 2021), Michigan, Minnesota, New Mexico, New York, Ohio, Oregon, Tennessee, and Utah.

© 2022 Havers FP et al. *JAMA Internal Medicine.*

Supplementary Figure. Selection of cases for analysis of adults ≥18 years with laboratory-confirmed COVID-19-associated hospitalization admitted January 1, 2021 – April 30, 2022, by vaccination status — COVID-NET, 13 States[a]

192,509 hospitalized adults ≥18 years admitted January 1, 2021–April 30, 2022, with positive SARS-CoV-2 test within ≤14 days of admission

↓

146,937 hospitalized adults ≥18 years linked to state vaccination registries (Vaccine Sample[b])

- Unvaccinated (n= 98,243)
- Partially vaccinated[c] (n= 9,062)
- Vaccinated with a primary series without a booster dose (n= 30,557)
- Vaccinated with a primary series with a booster dose (n=8,796)
- Vaccination status unknown (n= 279)

↓

14,164 hospitalized adults ≥18 years with chart review

→ Excluded **3,037** hospitalized adults ≥18 years

- Incomplete data (n= 184)
- Partially vaccinated [c] (n= 921)
- Vaccination status unknown (n= 16)
- Admitted primarily due to non-COVID-19-associated reasons[d] (1,916)
  - Unvaccinated (n=1,419), vaccinated (n=497)

↓

11,127 hospitalized adults ≥18 years admitted due to COVID-19 related illness (Comparison Sample)[e]:

- Unvaccinated (n= 8,575)
- Vaccinated with a primary series (n= 2,552)

↓

4,000 Propensity score matched cohort

- Unvaccinated (n= 2,000)
- Vaccinated with a primary series (n= 2,000)

[illegible]rveillance Network)

[a]California, Connecticut, Colorado, Georgia, Maryland (data excluded beginning December 4, 2021), Michigan, Minnesota, New Mexico, New York, Ohio, Oregon, Tennessee, and Utah

[b]Vaccine sample includes representative random sample from each site (sampled cases) as well as all non-sampled cases if all cases from a single site and age strata had vaccination status available.

[c]Received one dose of two-dose series (for Pfizer/BioNTech and Moderna) or had received the final dose of a primary series <14 days prior to positive SARS-CoV-2 test.

[d]Includes cases admitted due to OB/Labor and delivery admission (707), surgery (219), psychiatric admission needing acute medical care (270), trauma (290), "other" (190), and unknown reasons (240).

[e]Comparison sample includes only those sampled cases with complete medical chart abstraction who were either unvaccinated or vaccinated with a primary series; vaccinated cases who had evidence of prior SARS-CoV-2 infection prior to qualifying as completing a primary series were excluded.

NOTE: For both the vaccine sample and the analytic sample, sampling weights were created based on the probability of selection. Weights were adjusted for non-response, adjusted to population catchment totals based on combinations of surveillance site, time period of admission, age, sex, and race/ethnicity via published raking procedures, and trimmed to reduce variability, as previously described.[1]

© 2022 Havers FP et al. *JAMA Internal Medicine.*

© 2022 Havers FP et al. *JAMA Internal Medicine.*
